# CDK4/6 inhibition induces a senescence-associated secretory phenotype via delayed NF-κB activation

**DOI:** 10.26508/lsa.202603790

**Published:** 2026-07-09

**Authors:** Joanna Lan-Hing Yeung, Justin Rendleman, Lauren Anderson Westcott, Arnold Ou, Matthew Pressler, Nicole Pagane, Irene Duba, Ria Hosuru, Bat-Ider Tumenbayar, Viviana I Risca

**Affiliations:** Laboratory of Genome Architecture and Dynamics, The Rockefeller University , New York, NY, USA

## Abstract

Cellular senescence consists of regulated phenotypes associated with permanent exit from the cell cycle in response to stressors such as genomic instability. The consequences of senescence go beyond individual cells because of the senescence-associated secretory phenotype (SASP), which can induce inflammation in neighboring cells. Some cancers respond to CDK4/6 inhibitors (CDK4/6i) with a senescence-like phenotype in the absence of extensive DNA damage. We asked how the SASP and the transcriptional regulatory profile triggered by CDK4/6i-driven arrest compare with the canonical NF-κB–regulated SASP triggered by DNA damage. We profiled the dynamics of transcriptional regulation in response to the CDK4/6i, palbociclib, and the DNA-damaging agent, doxorubicin. We found that up-regulation of NF-κB–driven SASP genes was shared across both drugs, although delayed in CDK4/6i, coinciding with slower enhancer activation and epigenetic changes. ATM/ATR inhibition did not affect CDK4/6i-induced NF-κB nuclear localization, suggesting an alternative upstream activator. Inhibiting NF-κB suppressed the expression of SASP genes without reversing stable arrest, pointing to SASP manipulation as a potential therapeutic strategy and providing insights into controversies regarding cell cycle arrest–driven SASP.

## Abstract (precis)

Extended therapeutic inhibition of CDK4/6 pushes cancer cells into a senescent-like state and can trigger NF-κB activation to induce an inflammatory signature in both liposarcoma and ER+ breast cancer cells.

## Introduction

Cellular senescence is a state of stable cell cycle arrest that suppresses tumorigenesis by limiting the proliferation of cells that are aged, damaged, or stressed (Ajoolabady et al, 2025). However, even in established tumors, certain therapeutics can force cancer cells into therapy-induced senescence and thereby curb tumor growth (Schmitt et al, 2022). Although DNA damage has historically been considered a requirement for entry into senescence, emerging targeted therapies can induce senescent-like states outside of DNA-damaging agents and radiation; these include inhibitors of the cyclin-dependent kinases CDK4 and CDK6 (CDK4/6i) (Kovatcheva et al, 2017; Klein et al, 2018; Wagner & Gil, 2020). An outstanding question in the field is whether senescence, when caused by dramatically different triggers, converges on a common cell state, or represents a heterogeneous set of states sharing core phenotypic qualities. It is imperative to understand the molecular mechanisms underlying distinct growth arrest states, as regulatory networks intrinsic to normal aging and stress responses can ultimately shape therapeutic outcomes (Lee & Schmitt, 2019; Schmitt et al, 2022; Gleason et al, 2024).

Although senescent cells can no longer divide, they remain metabolically active and acquire features that continually evolve (Schmitt et al, 2022). As such, no universal biomarker of senescence exists, and identification of senescent cells relies on the characterization of several hallmarks (Hernandez-Segura et al, 2018). Beyond stable growth arrest, senescent cells exhibit enlarged morphology, heightened lysosomal content to maintain homeostasis in stressed conditions, increased nuclear size, and extensive alterations in the chromatin landscape, including senescence-associated heterochromatic foci (SAHFs) (Hernandez-Segura et al, 2018). Importantly, senescent cells secrete a complex network of cytokines and chemokines, along with other bioactive factors, known as the senescence-associated secretory phenotype (SASP) (Wang et al, 2024); this process increases the paracrine signaling of these “dormant” cells, thus imbuing them with significant influence over the neighboring environment.

Given its effects on the tumor microenvironment, identifying regulators of the SASP across distinct therapeutic triggers of senescence is critical for understanding its impact on disease outcomes. The SASP acts as a double-edged sword, having pro- and antitumorigenic effects, depending on its composition (Lau & David, 2019). The benefits of the SASP include reinforcing stable arrest through both autocrine and paracrine signals, while facilitating a robust antitumor immune response. In contrast, a chronic pro-inflammatory SASP drives increased invasive and migratory capacity of cancer cells, promoting angiogenesis and immune evasion (Schmitt et al, 2022).

The regulation and composition of the SASP have largely been investigated in model systems in which senescence is induced by DNA damage or genome instability (Rodier et al, 2009; Malaquin et al, 2016). Such triggers have been shown to induce the expression of inflammatory genes through stimulation of NF-κB, a master regulator of the SASP (Chien et al, 2011); this is coordinated via the DNA damage response kinase, ATM (Miyamoto, 2011; Bournique et al, 2025). Although DNA-damaging agents are frequently applied in cancer therapeutic approaches, targeted therapies like CDK4/6i can induce senescent-like states through non-DNA damage mechanisms. This begs the question, what drives the SASP when there is cellular senescence but not DNA damage? Prior studies of the response to CDK4/6i treatment have thus far offered conflicting results regarding the involvement of NF-κB (Goel et al, 2017; Guan et al, 2017; Wang et al, 2022; Gleason et al, 2024; Lee et al, 2024).

**Figure 1.**
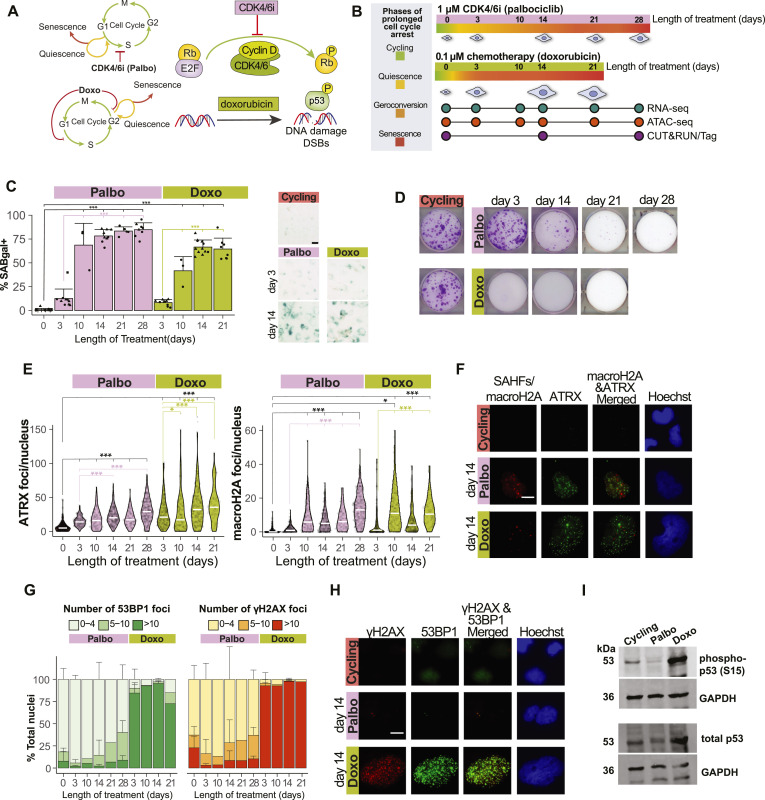
CDK4/6i and doxorubicin both induce senescence features with distinct levels of DNA damage and p53 activation. **(A)** Diagram summarizing CDK4/6i (Palbo) versus doxorubicin (Doxo) mechanisms of cell cycle arrest. **(B)** Schematic overview of comparative time course between CDK4/6i and doxorubicin and multi-omics approaches used to profile the transcriptome (RNA-seq), chromatin accessibility (ATAC-seq), and epigenome (CUT&RUN/Tag) in LS8817 cells. At least two biological replicates per timepoint and drug treatment were profiled. **(C)** Left: percent nuclei counted that had cellular SA-β-gal+ staining at different timepoints. The number of nuclei counted was >300 per condition at 10× objective magnification. Each point represents a field of view, with different shapes indicating biological replicates. Error bars denote SD. Right: representative SA-β-gal staining images at day 3 and day 14 shown at 10× objective magnification with scale bar = 50 μm **(D)**. Crystal violet staining after clonogenic outgrowth for 10–12 d. 800 cells were seeded per well in a 6-well plate in drug-free media after completion of drug treatment at each timepoint. **(E)** Number of ATRX foci (left) and macroH2A foci (indicative of SAHFs; right) per nucleus was counted for ∼100 nuclei per timepoint. **(F)** Representative ATRX and SAHF images are shown at day 14 of each drug treatment at 60× objective magnification. The scale bar is 15 μm. **(G)** Number of γH2AX foci and 53BP1 foci per nucleus was counted for ∼100 nuclei per timepoint and stratified into 0–4, 5–10, or >10 foci/nuclei. **(H)**. Representative images of γH2AX and 53BP1 staining are shown at day 14 of each drug treatment at 60× objective magnification. The scale bar is 15 μm. **(I)** Western blots of serine 15–phosphorylated p53 (phospho-p53) and total p53 in Cycling, Palbo-, and Doxo-treated LS8817 cell lysates harvested 18 d after treatment started. GAPDH was used as a loading control. Samples are from a separate experiment, independent of multi-omics data. Source data are available for this figure.

Here, we sought to investigate (1) does the presence (or absence) of DNA damage give rise to distinct therapy-induced senescent cell states and SASP profiles over time? and (2) what epigenetic changes dynamically regulate the SASP in cancer cells in response to therapeutics? We chose to address these questions systematically in a liposarcoma model, a rare type of malignant mesenchymal tumor with limited treatment options in advanced disease. Over 90% of liposarcoma tumors are characterized by co-amplification of *CDK4* and *MDM2* (Creytens et al, 2015), and previous work has shown that CDK4/6i can induce a full senescence program with irreversible arrest driven by *ANGPTL4*, correlating with patient outcomes and clinical efficacy (Kovatcheva et al, 2015; Dickson et al, 2016; Gleason et al, 2024). Although traditional chemotherapeutics, such as doxorubicin, trigger senescence through DNA damage, CDK4/6i instead arrest cells in G1 by preventing the activity of E2F transcription factors in a DNA damage–independent mechanism (Guan et al, 2017; Kovatcheva et al, 2017; Gleason et al, 2024; Fig 1A). We performed an extended time course directly comparing these two distinct triggers at timepoints after their induction of senescence (28 d of palbociclib versus 21 d of doxorubicin). By profiling epigenomic changes across multiple timepoints, we captured the temporal evolution of senescence-associated gene regulatory programs, including features that would be missed in short-term studies.

We find that early senescence programs are trigger-specific and largely remain distinct, including p53 activation after doxorubicin-induced DNA damage and an extracellular matrix (ECM)–enriched SASP program upon CDK4/6i treatment. At later timepoints, both therapies initiate a shared NF-κB–driven SASP, albeit with slower induction in the absence of DNA damage. Such activation of NF-κB is conserved beyond liposarcoma, observing a similar response in breast cancer cells. By extending the duration of CDK4/6i treatment here, we revealed delayed NF-κB activation and clarify its previously debated role from short-term studies, where its activity was not detected. Notably, NF-κB inhibition suppresses the shared SASP without reversing stable arrest, suggesting that selective modulation of SASP composition is possible while preserving growth arrest, representing new therapeutic opportunities to explore.

## Results

### CDK4/6i and doxorubicin both induce senescence features with distinct levels of DNA damage and p53 activation

To understand whether the absence or presence of DNA damage drives distinct cell states over time, we compared 1 μM palbociclib (Palbo), a type of CDK4/6i, against 100 nM doxorubicin (Doxo), standard chemotherapy, across multiple timepoints over a period sufficient to observe entry into senescence (28 d for palbociclib and 21 d for doxorubicin) in a patient-derived dedifferentiated liposarcoma cell line, LS8817 (Singer et al, 2007; Brill et al, 2010; Crago & Singer, 2011; Fig 1B). Although doxorubicin treatment elicited senescence phenotypes by even day 3 of treatment, we continued measurements through day 21 until enlarged cell size became prohibitive to continued culturing. Among multiple well-differentiated/dedifferentiated liposarcoma (WD/DDLS) cell lines tested, LS8817 was among those which undergoes stable senescence in response to palbociclib (Kovatcheva et al, 2015, 2017), providing a unique and experimentally tractable system for interrogating CDK4/6i-induced senescence in a clinically relevant cancer type. To investigate the full breadth of gene regulatory networks that underlie the senescent state, we performed a time-resolved, multi-omics analysis that profiles the transcriptome using mRNA-seq, accessible chromatin regions by ATAC-seq, and the epigenome using CUT&Tag and CUT&RUN methods (Fig 1B and Table S1).

Table S1. Quality control metrics for sequencing files.

We first validated that cells are entering into a senescent state in response to both drug treatments. Because there is no universal biomarker of senescence, we measured several accepted hallmarks of senescence. We observed an increased amount of lysosomal activity, through senescence-associated beta-galactosidase (SA-β-gal) staining, in both CDK4/6i and doxorubicin over time (Fig 1C). We confirmed cells entered a stable growth arrest state by assessing for clonogenic outgrowth upon drug removal (Fig 1D). In these clonogenic outgrowth assays, doxorubicin caused a stable arrest in nearly the entire population early in treatment, by day 3, whereas stable arrest did not occur for most cells in response to palbociclib until day 14 of treatment (Fig 1D). We also measured senescence-associated nuclear changes by immunofluorescence staining for the heterochromatin remodeler, ATRX (Kovatcheva et al. 2015, 2017), and the histone variant, macroH2A, a key structural SAHF component (Zhang et al, 2005; Fig 1E and F). Indeed, we saw that both treatments lead to an accumulation of both ATRX foci and SAHFs (Fig 1E and F), indicating that the global chromatin landscape does undergo dynamic changes during senescence.

Given the differences in the mechanisms of action for each drug, we anticipated the amount of DNA damage would be a distinctive feature between treatments. We measured the foci of γH2AX, a classical marker of DNA damage, and 53BP1, a DNA repair factor (Fig 1G and H). We found that even by day 28 of palbociclib treatment, the amount of DNA damage–associated foci remains comparable to that of untreated cycling controls (Fig 1G). This contrasts with doxorubicin treatment, under which DNA damage is robustly induced and remains elevated across all timepoints (Fig 1G). Next, we asked whether these differences in DNA damage are also reflected in the levels of phosphorylated p53, indicative of activation of the downstream DNA damage response (Sakaguchi et al, 1998; Saito et al, 2002; Loughery et al, 2014). Immunoblotting for phosphorylated p53 (serine 15) and total p53 protein levels confirmed that CDK4/6i treatment does not result in downstream activation of p53, whereas p53 is robustly activated in doxorubicin (Fig 1I). Thus, our results demonstrate that CDK4/6i treatment can induce senescence without the requirement for persistent DNA damage or activation of the DNA damage response.

### Global changes in the epigenome and transcriptome define drug-induced senescence trajectories

**Figure S1.**
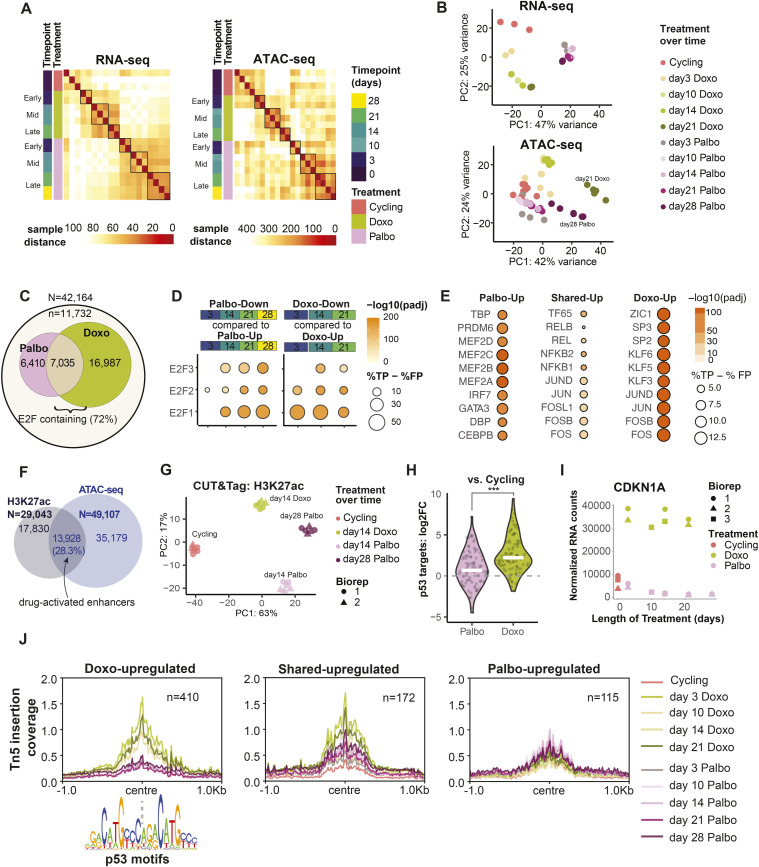
Global transcriptome and epigenome changes over time across drug treatments, related to Fig 2. **(A)** Distance measures between samples ordered by treatment and timepoint for RNA-seq (left) and ATAC-seq (right). **(B)** Principal component analysis on RNA-seq (top) and ATAC-seq (bottom) samples. **(C, D)** Venn diagram showing the number of peaks with decreased accessibility that contain E2F family of motifs relative to all down-regulated ATAC-seq peaks (D). Motif enrichment results of E2F family of motifs in down-regulated ATAC-seq peaks relative to up-regulated peaks (based on differential accessibility relative to Cycling) within each drug. The timepoints indicated represent the peak sets chosen as input for MEME-AME. Dot intensity represents −log10(Padj) values, whereas dot size reflects the difference between true-positive and false-positive peaks (%TP - %FP). **(E)** Motif enrichment results of top 10 most enriched motifs in ATAC-seq peaks up-regulated in palbociclib (Palbo-Up: left), both treatments (Shared-Up: middle), or doxorubicin (Doxo-Up: right), merged across all timepoints relative to Cycling and day 3, using unique up-regulated peaks from the alternate condition as a background. **(F)** Venn diagrams showing the number of up-regulated H3K27ac peaks that overlap with up-regulated ATAC-seq peaks, defined as drug-activated enhancers (see Methods). The color of text indicates the number of peaks from each peak set (H3K27ac: left; ATAC-seq: right). **(G)** Principal component analysis of H3K27ac CUT&Tag samples. **(H)** Log2 fold change (log2FC) values across all significantly changing p53 target genes in day 14 Palbo and day 14 Doxo, relative to Cycling. Paired *t* test shows difference between Palbo and Doxo is significant, with *P*-value = 4.468 × 10-8. **(I)** Normalized RNA-seq counts of the p53 target gene, *CDKN1A* (p21), over time. **(J)** Tn5 insertion sites around aggregated p53 motifs overlapping Doxo-, Shared-, or Palbo-up-regulated ATAC-seq peaks relative to Cycling (peaks from Fig 2A). The number of overlapping peaks with p53 motifs per condition is shown.

Given that both treatments drive cells into a senescent state, we asked whether the overall transcriptional regulatory state of these cells depends on the driver, particularly because doxorubicin triggers DNA damage, whereas palbociclib does not. We assayed the regulatory landscape with ATAC-seq to measure chromatin accessibility. Sample distance measures and principal component analysis showed that the global transcriptome and chromatin accessibility landscape continues to change throughout senescence (Fig S1A and B). These overall changes correspond to the timing of the stable growth arrest state as observed by accumulation of SA-β-gal and reduced clonogenic outgrowth (Fig 1C and D).

**Figure 2.**
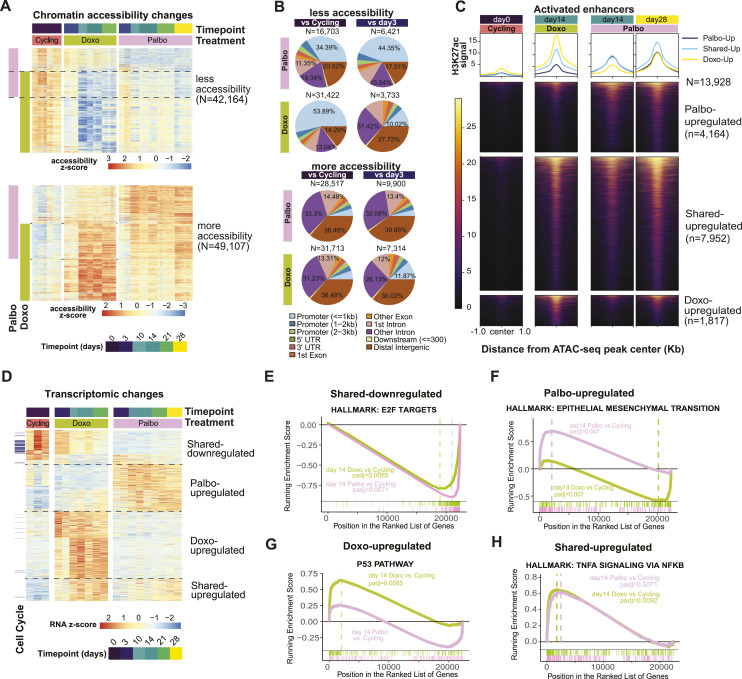
Distinct and shared transcriptional and chromatin responses define drug-specific senescence trajectories. **(A)** Heatmap of z-scores from VST-normalized read counts under ATAC-seq peaks with significantly less accessibility (top) or more accessibility (bottom) over time, relative to Cycling or day 3 treatment controls. Annotations indicate significance in Palbo (pink) or Doxo (green) and their genomic location. **(B)** Genomic feature annotation of ATAC-seq peaks with less accessibility (top) or more accessibility (bottom), merged across all timepoints compared with Cycling (left) or day 3 treatment controls (right). N = number of peaks in merged peak set making up the pie chart. **(C)** RPGC-normalized H3K27ac coverage in drug-activated enhancers (total number N = 13,928). Each row in the heatmap represents a drug-activated enhancer: defined as a region of increased chromatin accessibility overlapping a corresponding H3K27ac region increasing in palbociclib (Palbo-up-regulated), both treatments (Shared-up-regulated), or doxorubicin (Doxo-up-regulated). The number of regions plotted is shown on the right. The scale bar represents the bigWig signal of normalized coverage. **(D)** K-means clustered heatmap of z-scores from rlog-normalized counts for significantly changing genes across treatment and timepoints (days of treatment). Cell cycle genes are annotated in dark green and blue, respectively. **(E)** GSEA running enrichment score for enriched Hallmark term “E2F TARGETS” identified in Shared-down-regulated genes. Ranked gene list is based on day 14 Doxo and day 14 Palbo relative to Cycling comparisons. *Padj* values showing significance of enrichment are shown. **(F)** GSEA running enrichment score for enriched Hallmark term “EPITHELIAL MESENCHYMAL TRANSITION” identified in Palbo-regulated genes. **(G)** GSEA running enrichment score for enriched Hallmark term “P53 PATHWAY” identified in Doxo-regulated genes. **(H)** GSEA running enrichment score for enriched Hallmark term “TNFA SIGNALING VIA NFKB” identified in Shared-up-regulated genes. *Padj* values showing significance of enrichment are shown. Source data are available for this figure.

We found that many changes in chromatin accessibility trended in the same direction, but the timing and magnitude of the change were often dependent on the treatment (Fig 2A). A common feature among the senescent cells was decreased accessibility proximal to promoters (Fig 2B: top). These regions are enriched in E2F motifs (Fig S1C and D), and thus, their repression is indicative of cells disengaged from active cycling. Increases in accessibility, on the other hand, were predominantly localized to intergenic and intronic regions (Fig 2B: bottom), suggesting potential enhancer activation. These regions are enriched for motifs of stress-responsive and lineage-specifying transcription factors (Balamurugan & Sterneck, 2013; Kamachi & Kondoh, 2013; Sarkar & Hochedlinger, 2013; Golson & Kaestner, 2016; Tremblay et al, 2018; Huh et al, 2019; Romano & Miccio, 2020; Awasthi et al, 2021; Currey et al, 2021; Li et al, 2023; Ren et al, 2023; Fig S1E), consistent with activation of transcriptional programs that shape senescence-associated cell-fate decisions beyond cell cycle arrest.

We wondered whether enhancer activation dynamics differ depending on the timing of senescence entry; to capture these changes, we profiled H3K27ac, a histone mark of active enhancers. We found that 28% of all peaks that increased in accessibility also displayed significantly increased H3K27ac signal (Fig S1F), indicating remodeling of the enhancer landscape underlying the therapy-induced senescent state. Accordingly, we defined these regions as drug-activated enhancers (Fig 2C). Somewhat surprisingly, most of these activated enhancer elements were shared across treatment, albeit with different dynamics (Fig 2C). H3K27ac in shared-up-regulated enhancers gradually increases with CDK4/6i treatment, reaching levels similar to that of day 14 doxorubicin by day 28 (Fig 2C). This is supported by principal component analysis, which shows that day 28 palbociclib clusters more closely with day 14 doxorubicin than day 14 palbociclib (Fig S1G). This delayed enhancer activation in the CDK4/6i-treated cells corresponds to slower progression into stable growth arrest (Fig 1D).

We next asked how the transcriptional landscape compared between these two distinct therapy-induced senescence states, across multiple timepoints (Figs 1B and 2D). Consistent with decreased accessibility at promoter regions enriched in E2F motifs (Fig 2B: top, and Fig S1C and D), E2F target genes were down-regulated in both drug treatments, reflecting the cell cycle–arrested state (Fig 2D and E). We also observed more obvious drug-specific and temporal effects than at the chromatin accessibility and enhancer activation level (Fig 2D). This could be explained by differential transcription factor activity at DNA regulatory elements that are dependent on the DNA damage context. Among the genes that displayed higher expression levels in a drug-specific manner, palbociclib was related to a stronger “EPITHELIAL-TO-MESENCHYMAL TRANSITION” signature (Fig 2F). Phosphorylation of p53 at serine 15 has been shown to enhance p53 binding to chromatin, thereby facilitating transcriptional activation at p53-responsive promoters (Loughery et al, 2014). Supporting this notion, doxorubicin preferentially activated the p53 axis, as evidenced by *CDKN1A* induction of p53 target genes including *CDKN1A* (encoding p21) (Fig S1H and I), enrichment of the p53 pathway (Fig 2G) that was absent in palbociclib, and increased accessibility at p53 motifs (Fig S1J).

**Figure S2.**
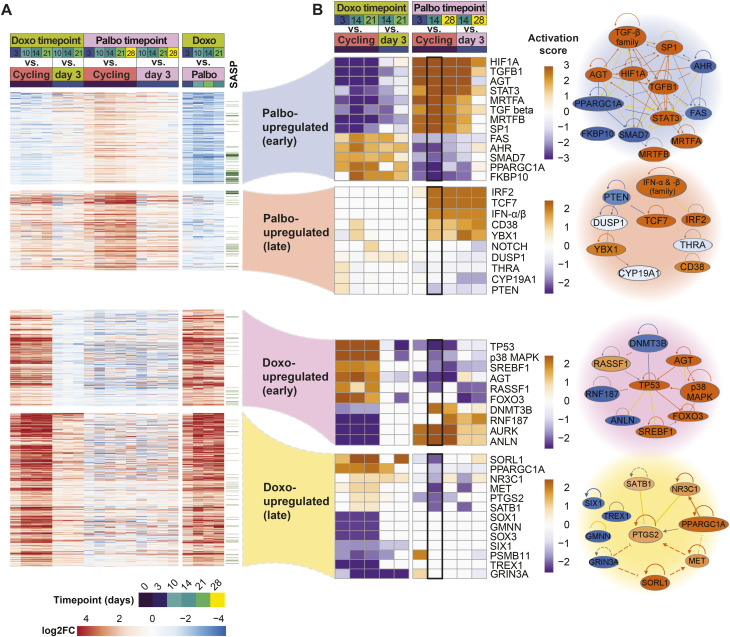
Predicted upstream regulators of drug-specific transcriptional responses, related to Fig 2. **(A)** Log2 fold change (log2FC) values of differentially up-regulated genes from Fig 2D, across different timepoints (days of treatment) and drug conditions, relative to Cycling or day 3 controls for each drug treatment. **(B)** Activation scores of the most differentially activated upstream regulators between Doxo and Palbo, corresponding to drug-distinct clusters of genes from Fig 2D. Differentially activated regulators were identified by summing activation scores across timepoints within each treatment and ranking them from highest to lowest (see Methods). A black outline marks the timepoint used to generate networks from IPA core analysis. Predicted relationships are shown (right) for each RNA cluster with legend at the bottom right indicating the confidence and type of predicted relationships.

Further analysis of drug-specific clusters revealed distinct early and late transcriptional responses within each treatment (Fig S2A). Notably, although some late-response genes were also up-regulated under the alternate condition, their induction was consistently greater in the treatment in which they were classified (Fig S2B: top). The early doxorubicin response was once again centered around p53 (Fig S2B: bottom), whereas the later response may involve metabolic adaptation through genes like *PPARGC1A* and *SORL1* (Fig S2B: bottom).

Although there were a large number of transcriptomic changes that were specific to the senescence trigger, we noted a set of genes that increased in expression as cells entered a senescent state independent of treatment (Fig 2D: Shared-up-regulated). This up-regulation was faster in the doxorubicin treatment and more gradual with palbociclib, coinciding with the dynamics of the enhancer activation we noted previously (Fig 2C). Gene set enrichment analysis (GSEA) revealed this set of genes was enriched for NF-κB transcriptional activity (Fig 2H), a motif we also observed among ATAC-seq peaks with shared increased accessibility between treatments (Fig S1E: middle).

### NF-κB is a predicted transcriptional regulator of the shared SASP in liposarcoma

Although multiple features of therapy-induced senescence vary between doxorubicin and palbociclib treatment responses (e.g., DNA damage activation, p53 signaling, and a large fraction of RNA and chromatin accessibility changes), we were particularly interested in what aspects these two very different drivers of stable arrest have in common. To investigate this, we focused on shared-up-regulated genes, which had distinct temporal dynamics in expression levels across drug treatments (Fig 2D), with a strong enrichment in the “TNFA SIGNALING VIA NFKB” Hallmark term (Fig 2H).

**Figure 3.**
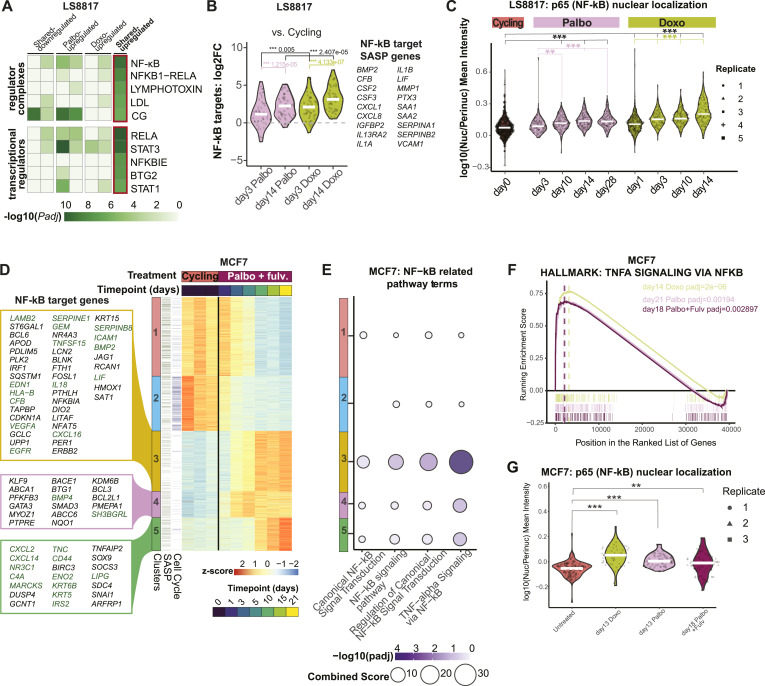
Shared senescence signature driven by NF-κB activation is delayed in CDK4/6i treatment across cancer cell types. **(A)** −Log10(*Padj*) values of top 5 IPA predicted upstream regulator complexes and transcriptional regulators of Shared-up-regulated genes relative to other RNA-seq clusters in LS8817 cells. **(B)** Left: Log2 fold change (log2FC) values of NF-κB target genes that are part of the Shared-up-regulated genes (RNA Cluster 7) in day 3 and day 14 Palbo and Doxo relative to Cycling in LS8817 cells. Median log2FC values for each condition are shown as a bold white line. The dotted gray line at log2FC = 0 indicates no change from Cycling. A paired *t* test was used to assess significance between log2FC distributions. Right: gene IDs of NF-κB target genes that are also defined as SASP genes based on whether they were classified as such in manually curated databases provided in Wang et al (2022) and Gleason et al. (2024) and the SASP Atlas (Basisty et al, 2020). **(C)** Quantification of log10 ratio of mean intensity of p65 in the nucleus to perinuclear region in LS8817 cells. Statistical significance was calculated using Dunnett’s test. Each point is a cell that was quantified. **(D)** K-means clustered heatmap of z-scores from rlog-normalized counts for significantly changing genes across treatment and timepoints (days of treatment) based on RNA-seq performed in MCF-7 cells. SASP and cell cycle genes are annotated in dark green and blue, respectively. NF-κB target genes found in each up-regulated gene cluster are shown left of the heatmap. Genes in dark green are also annotated as SASP. **(E)** GO enrichment results of NF-κB related terms from each RNA-seq cluster in MCF7 cells. **(F)** GSEA running enrichment score for enriched Hallmark term “TNFA SIGNALING VIA NFKB” for palbociclib, doxorubicin, and palbociclib+fulvestrant treatment in MCF7 cells. *Padj* values from GSEA are shown. **(G)** Quantification of log10 ratio of mean intensity of p65 in the nucleus to perinuclear region in MCF7 cells across different biological replicates. Statistical significance was calculated using Dunnett’s test. Source data are available for this figure.

**Figure S3.**
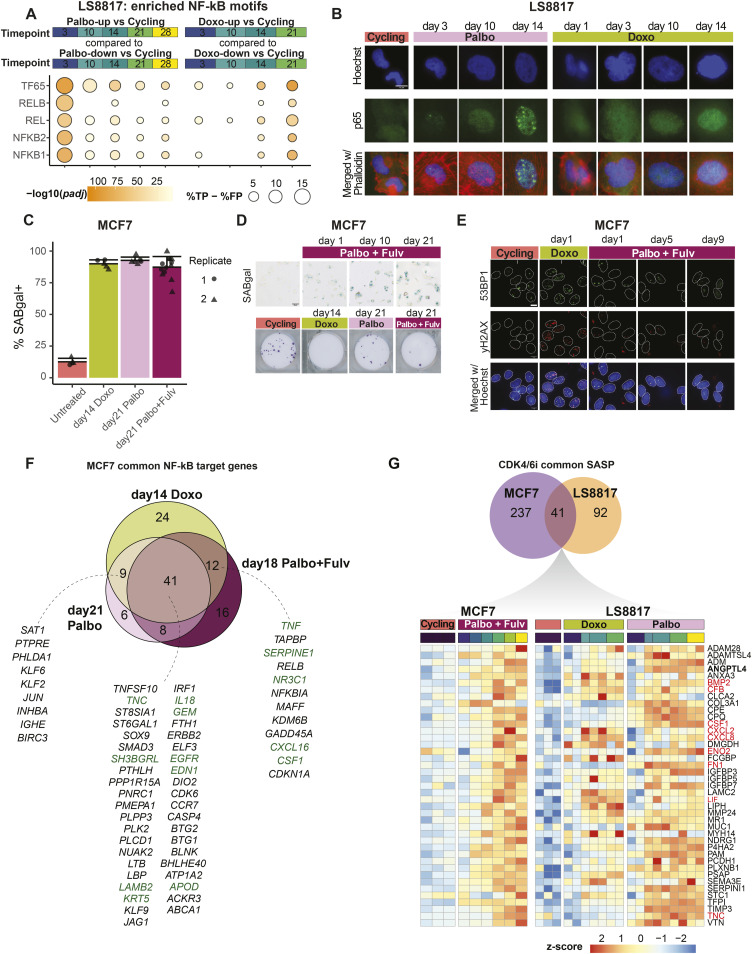
Additional evidence of NF-κB activity in LS8817 and MCF7 cells, related to Fig 3. **(A)** Motif enrichment results of NF-κB family of motifs in up-regulated ATAC-seq peaks relative to down-regulated peaks (based on differential accessibility relative to Cycling) within each drug. The timepoints indicated represent the peak sets chosen as input for MEME-AME. Dot intensity represents −log10 (*padj*) values, whereas dot size reflects the difference between true-positive and false-positive peaks (%TP - %FP). **(B)** Representative images of p65 immunofluorescence signal in LS8817 cells taken at 60× objective magnification throughout therapy-induced senescence. Cells were treated with 1 μM palbociclib (Palbo) continuously or 48 h with 100 nM doxorubicin (Doxo) before washout until harvest. The scale bar is 15 μm. **(C)** Percent nuclei counted that had cellular SA-β-gal+ staining in MCF7 cells for the following conditions: doxorubicin (Doxo), 1 μM palbociclib (Palbo), 100 nM palbociclib + 10 nM fulvestrant (Palbo + Fulv). The number of nuclei counted was >250 per condition at 20× objective magnification. Each point represents a field of view, with different shapes indicating biological replicates. Error bars denote the SD. **(D)** Crystal violet staining after 17 d of clonogenic outgrowth from 400 MCF7 cells seeded. **(E)** Representative images showing different levels of DNA damage (γH2AX foci & 53BP1 foci) in Doxo- and Palbo+Fulv-treated MCF7 cells over time. The scale bar is 10 μm. **(F)** Venn diagram showing overlap of up-regulated NF-κB target genes across conditions relative to untreated controls in MCF7. Gene IDs are indicated, with SASP genes highlighted in dark green. **(G)** Top: Venn diagram showing overlap of up-regulated CDK4/6-induced SASP genes common across LS8817 and MCF7 cells. Z-scores from rlog-normalized counts of CDK4/6-induced SASP genes commons across cancer cell types. SASP genes in red are also annotated as NF-κB target genes.

Despite its activity being undetected in prior short-term CDK4/6i treatment studies, the NF-κB complex and its canonical subunit, RELA (p65 or TF65), emerged as top predicted upstream regulators (Fig 3A). We identified 40 NF-κB targets within the shared-up-regulated gene module and analyzed their RNA levels at days 3 and 14 of treatment (Fig 3B). Approximately half of these genes were also defined as SASP across multiple databases (Fig 3B: right). Both drugs showed a significant increase in expression over time, consistent with a progressive NF-κB response (Fig 3B). However, this response was delayed in palbociclib-treated cells, with the median fold change at day 14 resembling that of doxorubicin at day 3 (Fig 3B). Corresponding to the shared induction of NF-κB target genes, NF-κB family motifs were similarly enriched in regions with increased chromatin accessibility in both drugs (Fig S3A). To confirm this shared NF-κB signature predicted by multi-omics analysis, we performed immunofluorescence on p65 (Figs 3C and S3B). In both treatments, we observe increased p65 nuclear localization, with timing that correlated approximately with the onset of stable cell cycle arrest (Fig 1D), that is, steadily increasing up to day 14 in response to palbociclib and much earlier, by day 3, in response to doxorubicin (Fig S3B).

### A shared NF-κB–driven SASP program is also found in MCF7 breast cancer cells

A previous study did not report NF-κB activation with 5 d of CDK4/6i treatment using the ER+ breast cancer model cell line, MCF7. Therefore, we wondered whether later timepoints would show NF-κB activation that could regulate the SASP in this system as well. To assess whether delayed NF-κB activation also occurred in this additional clinically relevant model, we treated MCF7 breast cancer cells with 1 μM palbociclib, 100 nM doxorubicin, or 100 nM palbociclib and 10 nM fulvestrant (Palbo+Fulv)—the standard-of-care regimen for ER+ breast cancer.

We confirmed that CDK4/6i induces senescence in MCF7 cells without triggering excess DNA damage, beyond what is observed in cycling cells, unlike doxorubicin (Fig S3C and E). We observed increased SA-β-gal staining (Fig S3C) and reduced clonogenic outgrowth (Fig S3D) across all conditions. Although strong stable arrest is observed in Palbo+Fulv, few γH2AX and 53BP1 DNA damage–associated foci accumulated compared with doxorubicin (Fig S3E), consistent with what we see in the liposarcoma context (Fig 1G and H).

Even in a model where NF-κB activity was previously undetected in short-term studies (Lee et al, 2024), we observed the eventual activation of NF-κB as a late treatment response. Time-resolved analysis over the course of 21 d of Palbo+Fulv treatment revealed 7,510 significantly altered genes based on a *Padj* threshold of <0.05 (Fig 3D). Strikingly, NF-κB–related pathways were enriched in a group of genes that are modestly up-regulated at early timepoints (days 3–5) but show strong induction after day 10 (Fig 3E, cluster 3). GSEA across different drug conditions in MCF7 samples showed that the “TNFA SIGNALING VIA NFKB” Hallmark term was significantly enriched in both palbociclib- and doxorubicin-treated samples (Fig 3F). Evidence of shared induction of NF-κB activity was further validated by immunofluorescence staining of p65, which showed increased nuclear localization across all drug treatments (Fig 3G). This supports the notion that NF-κB-SASP network activation is a shared feature of therapy-induced senescence across cancer types.

To identify shared NF-κB–associated SASP components, we examined NF-κB target genes up-regulated across treatments in MCF7 cells and found that 41 of 116 (35%) were common to palbociclib, Palbo+Fulv, and doxorubicin (Fig S3F). We next assessed conserved SASP programs driven by CDK4/6i across cell types. Several common SASP genes exist, including established NF-κB targets such as *CXCL2* and *CXCL8*, which were consistently up-regulated after CDK4/6i treatment in both MCF7 and LS8817 cells (Fig S3G). Although not a direct NF-κB target, *ANGPTL4*, a SASP factor necessary for CDK4/6i-mediated stable arrest (Gleason et al, 2024), was induced in both cell types. Moreover, *ANGPTL4* activates *IL1A*, a pro-inflammatory cytokine transcribed by NF-κB (Makulyte et al, 2026)—highlighting the complexity of the CDK4/6i-driven SASP, which may include components that can act either upstream or downstream of NF-κB. Together, these findings highlight a shared NF-κB response across distinct senescence-inducing treatments, alongside a CDK4/6i-specific SASP conserved across cancer cell types.

### Nearby enhancer activation contributes to the induction of shared NF-κB target genes

**Figure 4.**
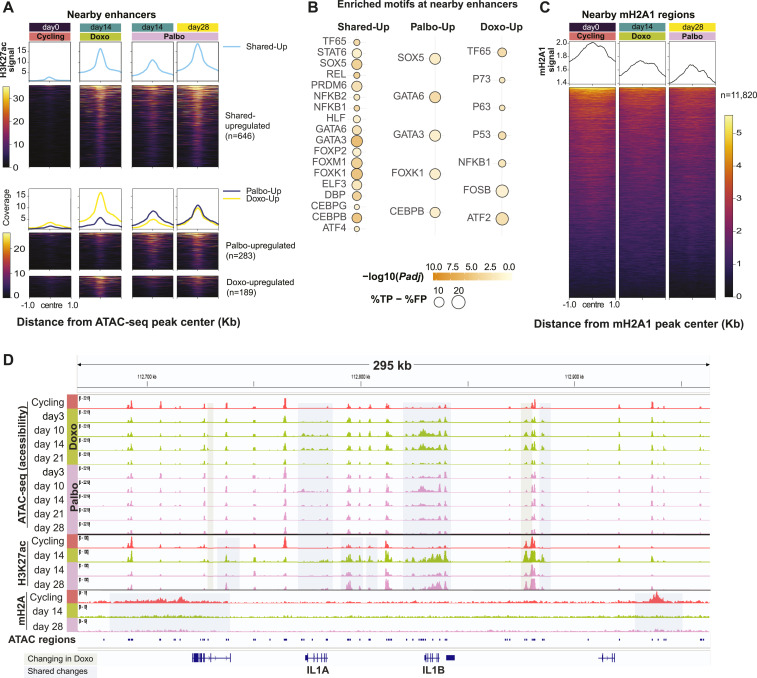
Activation and loss of macroH2A at enhancers near NF-κB–driven shared SASP genes. **(A)** H3K27ac coverage signal across conditions in drug-activated enhancers within 50 kb of shared-up-regulated genes (nearby enhancers). Each row in the heatmap represents a drug-activated enhancer: defined as a region of increased chromatin accessibility overlapping a corresponding H3K27ac region increasing in palbociclib (Palbo-up-regulated), both treatments (Shared-up-regulated), or doxorubicin (Doxo-up-regulated). The number of nearby peaks is shown on the left. Scale bar represents the bigWig signal of normalized coverage. **(B)**. Motif enrichment results of drug-activated enhancers shown in (A) within 50 kb of Shared-up-regulated genes. Dot intensity represents −log10 (padj) values, whereas dot size reflects the difference between true-positive and false-positive peaks (%TP - %FP). **(C)** macroH2A1 (mH2A1) signal for all mH2A1 peaks within 50 kb of shared-up-regulated genes (nearby mH2A1 regions) from Fig 2D. The number of nearby mH2A1 peaks is shown on the left. The scale bar represents the bigWig signal of normalized coverage. **(D)** Genome browser view of *IL1A* and *IL1B* loci. Normalized bigWig tracks for ATAC-seq, H3K27ac, and macroH2A1 (mH2A) are shown. Up-regulated regions occurring in both treatments are highlighted in blue (Shared changes), whereas Doxo-up-regulated regions are highlighted in green (Changes in Doxo). Source data are available for this figure.

Although NF-κB family motifs were globally enriched in regions of increased accessibility (Fig S3A), we sought to determine whether this enrichment was retained at regulatory elements more likely to directly influence expression levels of shared-up-regulated genes. To focus on such elements, we restricted our analysis to nearby up-regulated accessible regions, which were defined as within 50 kb of genes belonging to the shared-up-regulated gene module. Even under this constraint, we observed that increased Tn5 insertion coverage at TF65 (p65) motifs occurs across both drug treatments (Fig 4A and B).

Next, we identified nearby drug-activated enhancers by intersecting up-regulated accessible regions with sites of increased H3K27ac, specifically located within 50 kb of shared-up-regulated genes that increased in expression (Fig 4A). Notably, H3K27ac accumulation at these nearby enhancers was delayed in CDK4/6i relative to doxorubicin (Fig 4A: top). Coinciding with the slower kinetics of NF-κB target gene induction in CDK4/6i (Figs 2D and 3D), these shared-up-regulated enhancers proximal to shared-up-regulated genes were highly enriched in NF-κB family motifs, including canonical subunits TF65 and NFKB1 (Fig 4B: left).

In addition, motifs enriched in nearby palbociclib-up-regulated enhancers—including CEBPB, GATA3, GATA6, SOX5, and FOXK1 (Fig 4B: middle)—were also present at nearby shared-up-regulated enhancers (Fig 4B: top), suggesting continual engagement of transcription factors associated with mesenchymal enhancer activity and epithelial–mesenchymal transition programs. In contrast, doxorubicin-up-regulated enhancers near shared-up-regulated genes were in addition enriched for p53 family motifs (Fig 4B: right), consistent with activation of the DNA damage response.

**Figure S4.**
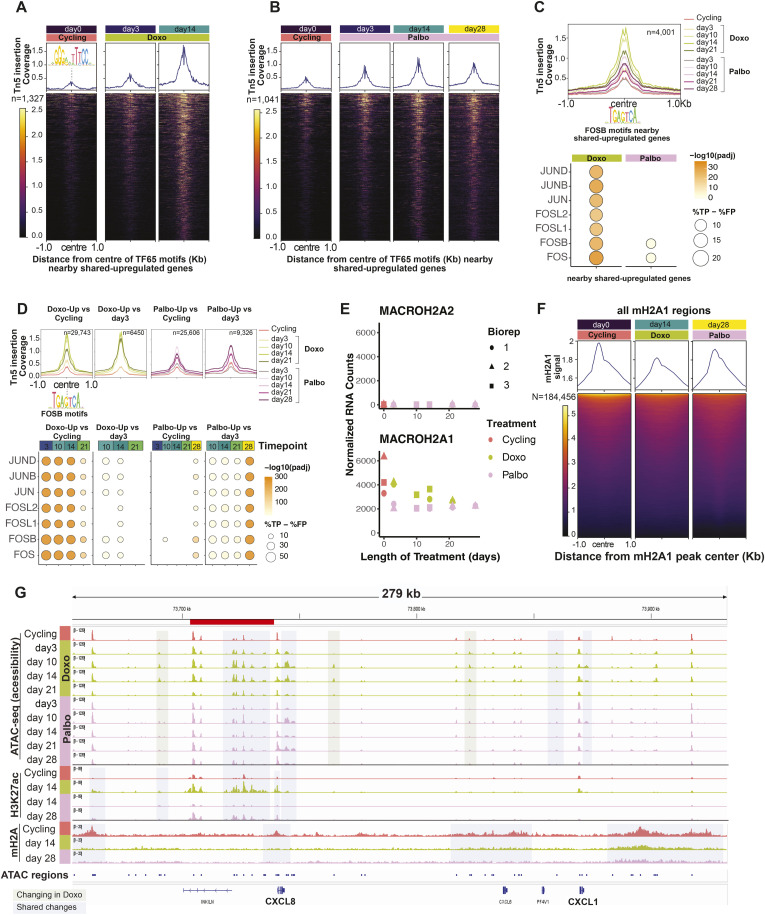
Additional ATAC-seq and epigenomic data, related to Fig 4. **(A)** Tn5 insertion site coverage at the center of the NF-κB subunit, TF65 (p65) motifs overlapping nearby Doxo-up-regulated ATAC-seq peaks, defined as within 50 kb of shared-up-regulated genes. The number of nearby peaks is shown on the left. The scale bar represents the bigWig signal of normalized coverage. **(B)** Same as (A), but for nearby Palbo-up-regulated ATAC-seq peaks. **(C) Top:** Tn5 insertion site coverage at the center of the AP-1 family member, FOSB motifs overlapping all up-regulated ATAC-seq peaks, in either drug treatment, within 50 kb of shared-up-regulated genes. **Bottom:** Motif enrichment results of AP-1 family of motifs (JUN/FOS) in up-regulated ATAC-seq peaks within 50 kb of shared-up-regulated genes (based on differential accessibility relative to Cycling or day 3 treatment controls) relative to nearby static peaks for each drug. The timepoints indicated represent the peak sets chosen as input for MEME-AME. Dot intensity represents −log10(Padj) values, whereas dot size reflects the difference between true-positive and false-positive peaks (%TP - %FP). **(D) Top:** Tn5 insertion site profiles around FOSB motifs overlapping all ATAC-seq peaks. ATAC-seq peaks increasing in accessibility at any timepoint in response to Doxo or Palbo, compared with Cycling or day 3 controls within each drug. The number of overlapping peaks with FOS motifs is shown. **Bottom:** Motif enrichment results of AP-1 family of motifs in up-regulated ATAC-seq peaks at each timepoint relative to Cycling or day 3 controls within each drug. **(E)** Normalized RNA-seq counts of *macroH2A2* and *macroH2A1* over time. **(F)** macroH2A1 (mH2A1) signal for all mH2A1 peaks called. The total number of mH2A1 peaks is shown on the left. The scale bar represents the bigWig signal of normalized coverage. **(G)** Genome browser view of *CXCL1* and *CXCL8* loci. Normalized bigWig tracks for ATAC-seq, H3K27ac, and macroH2A1 (mH2A) are shown. Up-regulated regions occurring in both treatments are highlighted in blue (Shared changes), whereas Doxo-up-regulated regions are highlighted in green (Changes in Doxo).

Interestingly, AP-1 motifs (e.g., FOSB and ATF2), a family of stress-responsive pioneer transcription factors (Biddie et al, 2011; Vierbuchen et al, 2017; Patrick et al, 2024), were also specifically enriched in doxorubicin-responsive enhancers near shared-up-regulated genes (Fig 4B: right). When considering regions gaining accessibility near the same genes, without requiring a change in acetylation, we found that there was some gain of accessibility at AP-1 motifs under palbociclib, but it was more modest than with doxorubicin (Fig S4C, top). Consistently, enrichment of AP-1 motifs in this accessible region set was also present at a low level (Fig S4C, bottom). However, when considering all up-regulated ATAC-seq peaks genome-wide, peaks containing AP-1 motifs were strongly enriched at all times under doxorubicin treatment but also significantly enriched under palbociclib treatment, especially after day 3—exhibiting delayed activation kinetics (Fig S4D). Given that AP-1 has been identified as a master regulator of oncogene-induced senescence (Martínez-Zamudio et al, 2020), a DNA damage–driven process, the combined enrichment of p53 and AP-1 motifs at doxorubicin-up-regulated enhancers may contribute to the earlier and more robust activation of NF-κB compared with CDK4/6i.

### Loss of macroH2A is associated with increased expression of NF-κB–driven shared SASP genes

In addition to enhancer activation, we wondered whether other nearby changes in the epigenome contributed to activation of NF-κB–driven SASP genes. It is thought that the spatial rearrangement of heterochromatin into SAHFs plays a role in silencing cell cycle progression genes (Narita et al, 2003), while allowing for SASP genes to be sequestered into a transcriptionally permissive environment (Aird et al, 2016). Given the role that the histone variant, macroH2A, plays in transcriptional repression and the formation of the SAHFs (Zhang et al, 2005, 2007), we used CUT&RUN to map the genome-wide distribution of macroH2A1 in late-stage therapy-induced senescence. We find that macroH2A2 is not expressed in this cell line (Fig S4E: top); in addition, neither macroH2A1 RNA levels (Fig S4E: bottom) nor its global distribution on chromatin changed with drug treatment (Fig S4F). However, by examining macroH2A1-containing regions found within 50 kb of shared-up-regulated genes, we observe a striking loss of macroH2A (Fig 4C). Hence, in addition to enhancer activation, locally reduced levels of macroH2A may also contribute to the activation of inflammatory SASP genes, likely through a derepression mechanism.

We also focused on chromatin changes surrounding specific canonical SASP genes up-regulated across both treatments. We examined a ∼300-kb window around the *IL1A, IL1B* (Fig 4D), *CXCL1*, and *CXCL8* loci (Fig S4G). Across both treatments, we observed shared increases in chromatin accessibility as early as day 3, spanning gene bodies, transcription termination sites, and distal intergenic regions (Figs 4D and S4G). Despite these early accessibility gains, H3K27ac accumulation was delayed, remaining lower at day 14 in CDK4/6i-treated cells compared with doxorubicin before gradually increasing to comparable levels by day 28 (Figs 4D and S4G).

Finally, we identified enhancers selectively activated in doxorubicin-treated cells, which may contribute to the earlier and more robust induction of shared SASP genes observed under this condition (Figs 4D and S4G). Compared with ATAC-seq and H3K27ac signal, macroH2A1 coverage is broader and more diffuse, with signal depletion generally occurring in regions flanking—rather than within—the gene bodies of these inflammatory loci (Figs 4D and S4G). This pattern is surprising and suggests an additional regulatory layer beyond the transcribed gene-body macroH2A depletion predicted by the “transcriptional pruning” model (Sun et al, 2018).

### The NF-κB–driven shared SASP component can be uncoupled from stable cell cycle arrest

**Figure 5.**
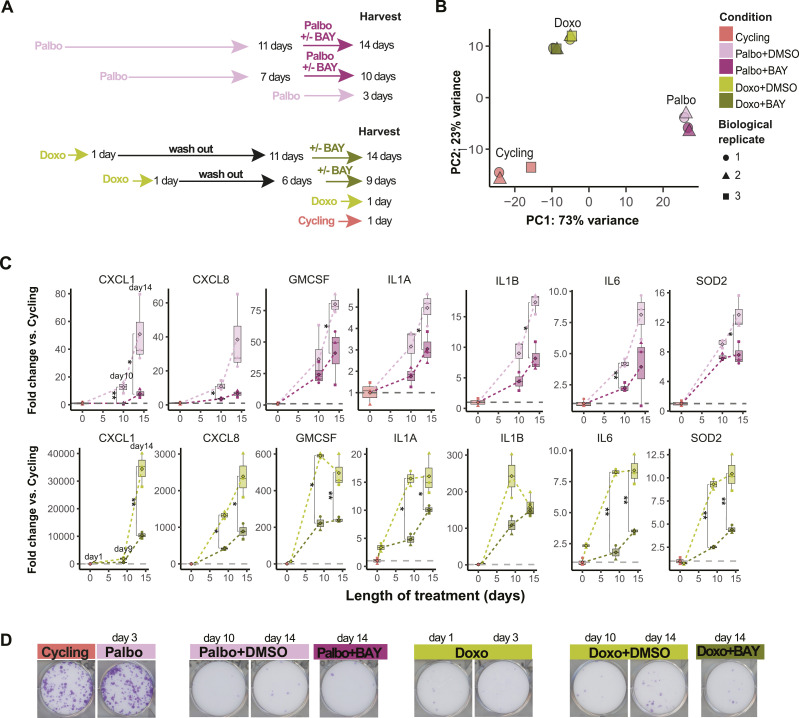
Inhibition of NF-κB activity suppresses the shared SASP without reversing stable growth arrest. **(A)** Schematic of pharmacological inhibition of NF-κB with BAY11-7082 (BAY) in LS8817 cells (see Methods). 10 μM BAY or equivalent volume of DMSO (negative control) was added to cells for 2–3 d before harvest. **(B)** Principal component analysis on RNA-seq of day 14 palbociclib- or doxorubicin-treated LS8817 cells with or without NF-κB inhibitor, BAY11-7082 (BAY). **(C)** RT–qPCR results of palbociclib- or doxorubicin-treated LS8817 cells with or without BAY. Relative fold change values to Cycling are shown. A dotted gray line indicates no change. Diamonds inside boxplot represent the mean fold change value. Circles, triangles, and rectangles represent the fold change of individual biological replicate. Dark shades indicate Palbo and Doxo samples treated with BAY, whereas lighter shades indicate samples that had DMSO added at the same time as BAY samples. Statistical significance was calculated using a two-tailed *t* test. **(D)** Crystal violet staining after clonogenic outgrowth after palbociclib or doxorubicin treatment ±BAY. 800 cells were seeded per well in a 6-well plate and grown in drug-free media for 12 d after harvest. Source data are available for this figure.

**Figure S5.**
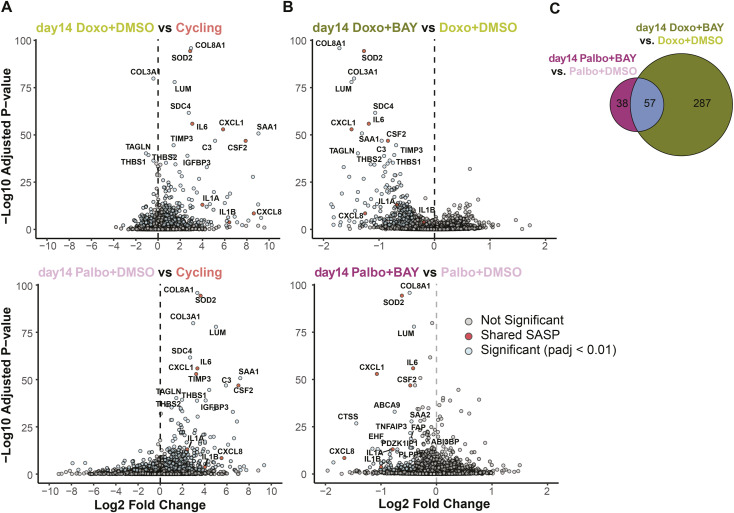
BAY-11-7082 treatment suppresses the up-regulation of NF-κB target genes in LS8817 cells, related to Fig 5. **(A)** Volcano plot from RNA-seq analysis showing shared NF-κB–driven SASP genes and other top 15 most significantly changing genes in day 14 palbociclib or doxorubicin treated with DMSO control. **(B)** Same as (A) but for day 14 palbociclib or doxorubicin treated with BAY. **(C)** Venn diagram showing the number of genes down-regulated by BAY relative to day 14 palbociclib- or doxorubicin-treated DMSO controls.

We next considered whether NF-κB activation was necessary for cells to drive stable arrest, or is it solely responsible for the shared SASP program without any additional effects on senescence? We attempted to alter the NF-κB–driven shared SASP response through pharmacological inhibition with BAY11-7082 (BAY) (Pierce et al, 1997) during both treatments, after senescence was established (Fig 5A). Principal component analysis shows NF-κB inhibition had a minimal impact on the overall transcriptome, as BAY- and DMSO-treated samples clustered together within each drug group (Fig 5B). However, BAY suppressed the expression of key shared NF-κB target SASP genes, *CXCL1, CXCL8, IL1A, IL1B, GMCSF* (*CSF2*), *IL6*, and *SOD2* (Figs 5C and S5A and B).

Beyond these, NF-κB inhibition down-regulated 57 shared genes, as well as additional drug-specific targets (Fig S5C), suggesting that although NF-κB is a common feature of senescence, its ability to regulate specific targets may differ across treatments. In addition, some gene expression changes outside of the expected NF-κB targets may represent off-target effects of the BAY treatment; future validation with genetic perturbations could help to differentiate these instances. Like previous studies of NF-κB in other types of senescence (Chien et al, 2011; Lau & David, 2019), inhibiting its activity was not enough to reverse senescence, as indicated by no differences in clonogenic outgrowth assays (Fig 5D). Hence, although NF-κB activation does not appear to be necessary for maintaining stable arrest, it is responsible for driving part of the inflammatory SASP response.

### DNA damage response pathway is upstream of NF-κB nuclear localization in doxorubicin but not CDK4/6i

NF-κB activity can be triggered by a variety of cellular stressors, among which the DNA damage response (DDR) is a major contributor (Miyamoto, 2011). Notably, several contexts, such as mitochondrial dysfunction and treatment with histone deacetylase inhibitors, have demonstrated that senescence-associated DDR signaling can be engaged in the absence of overt DNA damage (Pospelova et al, 2009; Pazolli et al, 2012; Wiley et al, 2016).

**Figure S6.**
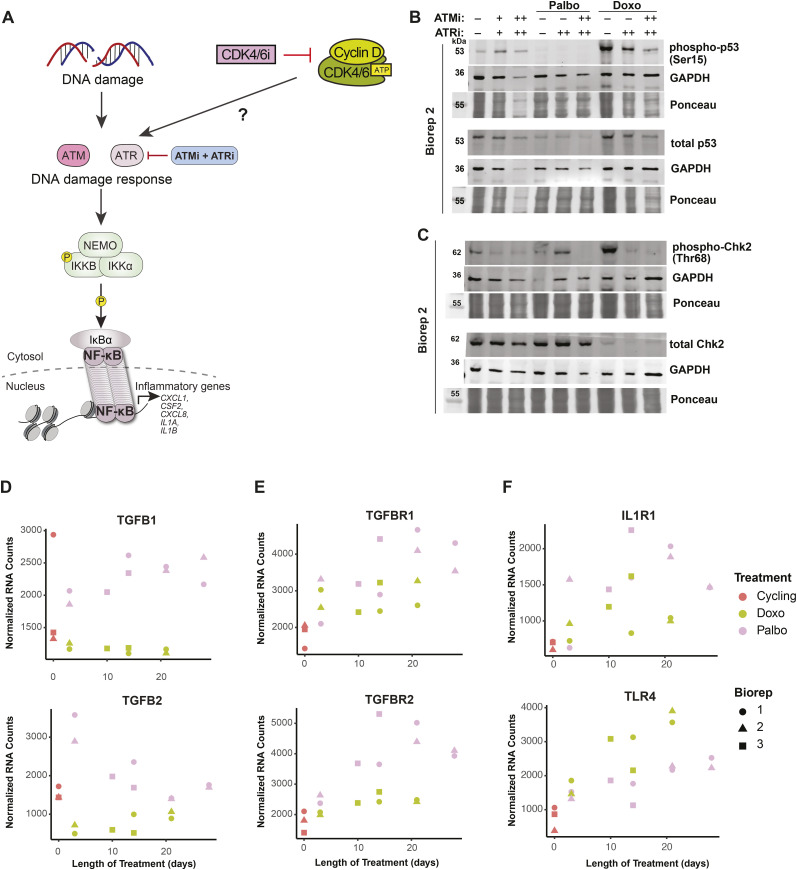
Up-regulation of cell-surface receptors upstream of NF-κB activation is found in CDK4/6i treatment, related to Fig 6. **(A)** Schematic of the experimental strategy testing whether inhibition of the DNA damage response kinases ATM and ATR affects NF-κB activation in CDK4/6i treatment in LS8817 cells. **(B)** Second biological replicate of Western blots for phospho-p53 and total p53, corresponding to Fig 6A. **(C)**. As in (B), but for phospho-Chk2 and total Chk2. **(D)** Normalized RNA-seq counts of *TGFB1* and *TGFB2* over time. **(E)** Normalized RNA-seq counts of *TGFBR1* and *TGFBR2* over time. **(F)** Normalized RNA-seq counts of *IL1R1* and *TLR4* over time.

**Figure 6.**
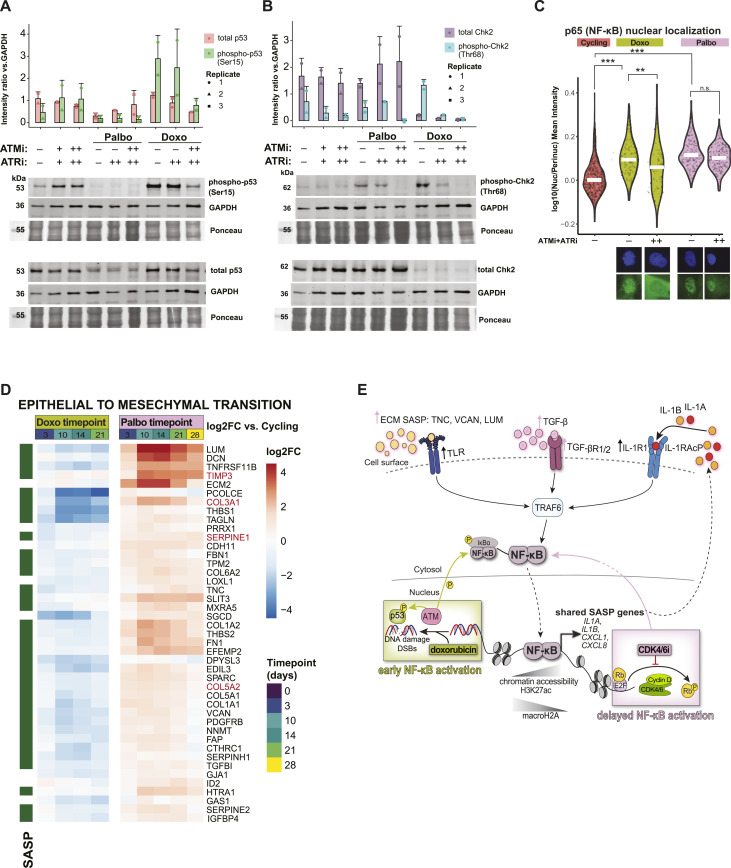
Inhibiting ATM and ATR affects NF-κB nuclear localization in doxorubicin but not CDK4/6i therapy-induced senescence. **(A)** Western blots of LS8817 cells treated with Palbo (17 d) or Doxo (7 d), with or without ATR inhibition (ATRi; 4 μM AZD6783) or combined ATR and ATM inhibition (ATRi + ATMi: 4 μM AZD6783 + 4 μM KU-60019) for two biological replicates (Bioreps). ATR and ATM inhibitors were added after cell cycle arrest had been established. ImageJ quantification of signal relative to the GAPDH loading control (top). Phospho-p53 (Ser15) relative to total p53 (bottom). “–” indicates DMSO control; “+” and “++” indicate 2 and 4 μM inhibitor, respectively. Replicate indicates different biological replicates. **(B)** Same as (A) but Western blots for phospho-Chk2 (Thr68) relative to total Chk2. **(C)** Quantification of log10 ratio of mean intensity of p65 in the nucleus to perinuclear region in LS8817 cells treated with ATMi+ATRi or DMSO. Statistical significance was calculated using Dunnett’s test. Representative images taken at 60× objective magnification of different amounts of p65 nuclear localizations in each condition are shown at the bottom. **(D)** Log2 fold change (log2FC) values of Palbo-up-regulated genes that are under the Hallmark term “EPITHELIAL-TO-MESENCHYMAL TRANSITION,” across different timepoints (days of treatment) and drug conditions, relative to Cycling or day 3 controls for each drug treatment. Genes that are also SASP are shown on the left in dark green. Genes that are also NF-κB targets are shown in red text. **(E)** Summary model showing possible pathways activated by CDK4/6i and doxorubicin to induce the shared SASP driven by NF-κB at different temporal rates. Source data are available for this figure.

Although CDK4/6 inhibition does not directly induce DNA damage, these previous studies raise the possibility that DDR components could nonetheless be engaged through other mechanisms. To test whether DDR signaling contributes to NF-κB activation in this context, we pharmacologically inhibited the key DDR kinases ATM and ATR in arrested LS8817 cells (Fig S6A). We confirmed that the DDR signaling was dampened by ATM and ATR inhibition by immunoblotting for phosphorylated (phospho-) p53 (Figs 6A and S6B) and Chk2 (Figs 6C and S6C). Phospho-p53 is largely absent after palbociclib treatment, and co-treatment with ATM and ATR inhibitors did not alter phospho-p53 levels (Fig 6A). Phospho-Chk2 levels were modestly increased under palbociclib treatment, suggesting mild ATM activation in the absence of p53 activation and additional DNA damage (Fig 6B). In contrast, doxorubicin treatment induced robust p53 and Chk2 phosphorylation and activation, which was attenuated by ATM and ATR inhibition (Fig 6A and B). This corresponded to reduced p65 nuclear localization, consistent with DNA damage–dependent activation of NF-κB (Fig 6C). In contrast, inhibiting ATM and ATR did not affect NF-κB nuclear localization in CDK4/6i-treated cells (Fig 6C), indicating that NF-κB activation in this setting occurs independently of the canonical DDR pathway.

### ECM-driven SASP is specifically activated in CDK4/6i-induced senescence

Given that inhibition of DNA damage response factors did not affect NF-κB nuclear localization in CDK4/6i-treated cells, we next sought to identify alternative upstream pathways by characterizing gene expression programs specific to CDK4/6i-induced cell cycle arrest. Notably, genes associated with TGF-β signaling, including ligands (Fig S6D) and receptors (Fig S6E), were selectively up-regulated in CDK4/6i-treated cells, but not in doxorubicin-treated cells. In addition, many of these genes encode ECM components and established TGF-β targets, several of which have been identified as SASP genes in other contexts (Fig 6D). These findings suggest that in parallel to the later NF-κB–driven shared SASP, CDK4/6 inhibition promotes an earlier, ECM-enriched SASP program.

Consistent with a role of extracellular signaling in this context, *IL1R1*, the primary receptor that *IL1A* and *IL1B* bind to (Wesche et al, 1997), increases in expression during CDK4/6i treatment (Fig S6F: top). This indicates potential activation of cell-surface receptors that could drive downstream NF-κB activation. We also detected increased expression of *TLR4* in both treatment conditions (Fig S6F: bottom), pointing to pattern recognition receptor signaling as an additional route to NF-κB activation (Kelsh & McKeown-Longo, 2013; Guo et al, 2024). We hypothesize that up-regulation of multiple cell-surface receptors—including *TGFBR1/2, IL1R1*, and *TLR4* (Fig S6D–F)—along with ECM-enriched SASP components (Fig 6D) that engage these receptors may provide alternative routes to NF-κB activation in CDK4/6i-induced senescence, independent of intrinsic DNA damage signaling (Fig 6E).

## Discussion

Therapeutic response in various cancers is in part determined by how effectively tumors are driven into stable growth arrest. However, even upon entering such a senescent state, the profile of secreted molecules, or SASP, plays an important role in influencing the neighboring environment (Wang et al, 2024). Unlike replicative senescence that arises over a lifetime (McHugh & Gil, 2018), therapy-induced senescence-like states are acute responses to cancer treatments, exhibiting many features of canonical senescence although mediated through distinct mechanistic triggers (Wagner & Gil, 2020; Wang et al, 2020; Schmitt et al, 2022). Such acute models of senescence offer new insights into the underlying networks that are accessed during these processes and assist us in revealing common underlying features of the elusive core components of what it means to be a senescent cell. In addition, understanding how the composition of the SASP changes over time across different cancer therapies will be essential in identifying new targeted approaches that improve patient outcomes.

Here, we have used a dedifferentiated liposarcoma patient–derived cell line known to enter senescent-like states in response to CDK4/6i, and identified NF-κB as a key regulator of the common SASP inflammatory response. We show that this module of the response arises despite being triggered by a DNA-damaging agent (i.e., doxorubicin) or a DNA damage–independent mechanism (i.e., the CDK4/6 inhibitor palbociclib), and can be decoupled from stable arrest. We confirm this shared NF-κB activation also occurs in an estrogen receptor–positive (ER+) breast cancer cell line model (MCF7), supporting the generality of this regulatory node as a key feature of therapy-induced SASP. Although the use of cell line models does not allow for the assessment of the SASP’s effects on immune clearance and/or evasion, we sought to focus on the molecular landscape of the tumor cell to clarify this context. Therefore, we mapped the temporal dynamics of epigenomic and transcriptomic networks at different phases of the CDK4/6i-induced senescence, by analyzing the genome-wide chromatin accessibility, H3K27ac-marked enhancers, and mRNA levels at early-quiescent (day 3), mid-transition (day 14), and late-senescent (day 28) timepoints (Fig 1B). We compared this with the DNA damage–induced senescent state that arises rapidly upon doxorubicin exposure.

Most transcriptomic changes are drug-distinct differences explained by p53 activation specific to DNA damage induced by doxorubicin (Fig 2D and G) and a CDK4/6i-specific treatment response characterized by the increased expression of ECM-related genes (Figs 2D and F and S2B). Nonetheless, we identified a common SASP-associated enhancer module enriched for NF-κB motifs that displayed delayed activation in CDK4/6i relative to doxorubicin, which was further substantiated by later accumulation of H3K27ac at enhancer elements (Figs 2B, 3A and B, and 4A and B). A concurrent loss of the SAHF component macroH2A around these loci may further contribute to the induction of the shared SASP module (Fig 4C). Although loss of macroH2A has been linked to derepression of inflammatory loci in oncogene-induced senescence (Chen et al, 2015), to our knowledge this is the first report of its depletion in CDK4/6i-induced senescence, although its functional role here remains to be determined.

Our data reveal a previously underappreciated inflammatory component of CDK4/6i-induced senescence, with potential implications for long-term therapeutic outcomes. Although NF-κB’s role in senescence induced by oncogene activation is established (Chien et al, 2011; Salminen et al, 2012), its activation in CDK4/6i-induced senescence was surprising, as prior studies failed to detect NF-κB activity (Goel et al, 2017; Wang et al, 2022; Lee et al, 2024). This discrepancy likely reflects differences in the duration of treatment, as these studies were focused on early responses. We did not detect NF-κB activation in earlier timepoints, in line with a previous observation in MCF7 cells after five days of treatment (Lee et al, 2024). However, by increasing the duration of CDK4/6i treatment (i.e., 14, 21, 28 d) we observed delayed but robust NF-κB activation across both liposarcoma and breast cancer cells.

To determine whether the NF-κB–driven inflammatory response is necessary for the cell to enter stable arrest, we showed that important shared SASP genes (*CXCL1, CXCL8*, *IL6*, *IL1A*, and *IL1B*) can be effectively suppressed by NF-κB inhibition *without reversing stable arrest* (Fig 5A–D). Many of these shared SASP factors have known tumor-promoting functions, including immunosuppression, metastasis, and chemoresistance (Ancrile et al, 2007; Acosta & Gil, 2009; Jones & Jenkins, 2018; Lau & David, 2019; Rebe & Ghiringhelli, 2020; Garlanda & Mantovani, 2021; Wu et al, 2022). The SASP may therefore be tunable by adjuvant therapies to diminish protumorigenic side effects. One caveat in the current study that warrants future investigation is our use of pharmacological inhibition of NF-κB rather than genetic perturbation. Although general RNA levels are not impacted over the course of treatment to the degree that they would dramatically alter the transcriptomic state within each drug treatment (Fig 5B), BAY11-7082 treatment could impact other unrelated pathways, including altering the degradation rates of proteins through off-target effects on deubiquitinates. Therefore, it is an important consideration especially regarding specific genes identified by our BAY experiments and assessing whether they are direct targets of NF-κB or are among potential off-target effects of the inhibitor.

Beyond shared targets, CDK4/6i also induces a distinct ECM-enriched SASP program (Fig 6D) that may be driven by TGF-β signaling (Figs S2B and S6D and E) and its downstream transcription factors (Fig 4B). Future work is needed to understand the interplay and regulation between CDK4/6i-distinct and shared components of the SASP. Nonetheless, our data support a growing shift in the way we think about the clinical implications of CDK4/6i treatment. Although it may offer initial benefits to patients over traditional chemotherapy, over time it leads to the emergence of a pro-inflammatory SASP driven by NF-κB that can promote tumor progression. Further therapeutic options that modulate the SASP while maintaining arrest are an area ripe for development.

These findings improve our understanding of the questions we set out to answer regarding the dependence of the SASP on DNA damage and the dynamic nature of the epigenetic changes that underlie the response. Because macro was previously shown to activate enhancers in response to CDK4/6i in breast cancer (Watt et al, 2021), we asked whether increased accessibility at AP-1 motifs contributed to the differences in the temporal activation of the SASP. In our data, both drug treatments showed evidence of AP-1 activation but exhibited distinct kinetics: rapidly increased in doxorubicin and more gradual with CDK4/6i (Fig S4C and D), with additional AP-1 enrichment at Doxo-specific increased H3K27ac regions. This may indicate that doxorubicin triggers full enhancer activation at AP-1 bound sites, whereas palbociclib treatment merely causes AP-1 factors to open chromatin via pioneering activity (Biddie et al, 2011; Vierbuchen et al, 2017). AP-1 is known to act synergistically with NF-κB to drive the expression of its target genes through genome-wide chromatin remodeling (Fujioka et al, 2004; Oeckinghaus et al, 2011; Ji et al, 2019), which offers an explanation for the timing and magnitude differences of NF-κB–driven SASP activation across treatments; earlier and robust in doxorubicin but delayed in CDK4/6i.

An outstanding question is what drives NF-κB activation in CDK4/6i-induced senescence in the absence of DNA damage? Inhibition of ATM and ATR did not affect NF-κB nuclear localization with CDK4/6i but indeed blocks this response with doxorubicin (Fig 6C), supporting a DNA damage response–independent mechanism in the CDK4/6i context. Although we chose to inhibit ATM and ATR after cell cycle arrest to assess their impact as cells transition from a quiescent to senescent state at which point they engage a SASP program, this does leave the possibility that DNA damage occurring before our inhibition may contribute to the activation of NF-κB in an earlier window, whose effect may then persist. However, taking our data as a whole, we would propose an alternative model for NF-κB activation in CDK4/6i treatment that is mediated through alternative upstream pathways, which we speculate could include TGF-β and/or IL-1, known mediators of this pathway (Guo et al, 2024). Both *TGFBR1/2* and *IL1R1* expression levels are up-regulated in CDK4/6i treatment (Fig S6D–F), and converge on the TRAF6-TAK1 signaling axis, resulting in nuclear localization of NF-κB (Freudlsperger et al, 2013; Wang et al, 2021). In addition, both CDK4/6i and doxorubicin treatment cause increased expression of *TLR4*, another cell-surface receptor that feeds into NF-κB activation (Guo et al, 2024). *TLR4* can be activated by endogenous damage-associated molecular patterns (DAMPs), including ECM components, versican (*VCAN*) and tenascin C (*TNC*) (Kelsh & McKeown-Longo, 2013; Han et al, 2020), which are part of the CDK4/6i-enriched SASP (Fig 6E). Activation of NF-κB promotes continued up-regulation of *IL1A* and *IL1B* (Figs 3B and 4D)*,* resulting in a positive feedback loop that sustains NF-κB activity (Croston et al, 1995; Weber et al, 2010). Together, our data suggest a model to be tested in future studies (Fig 6E), whereby NF-κB activation in CDK4/6i-treated cells arises gradually through up-regulation of cell-surface receptors and extracellular signaling rather than acute DNA damage.

Our results underscore two important considerations: (1) the importance of the underlying chromatin landscape that can vary intrinsically by cell type and extrinsically by trigger response, and (2) the dynamic and tunable nature of the SASP. In conclusion, we propose a model in which both doxorubicin and CDK4/6i induce senescence through distinct molecular mechanisms that converge over time, leading to NF-κB activation through enhancer remodeling and loss of macroH2A. These epigenetic changes promote a shared senescence response that manifests as a common set of cytokines and chemokines, which are secreted as part of the SASP. Our results bring new insights that help explain conflicting reports on various features of therapy-induced senescence, including the involvement of NF-κB across two therapeutics with dramatically different DNA damage profiles and activation dynamics.

## Materials and Methods

### Cell lines and cell culture

A human LS8817^TetON FLAG-MDM2^ liposarcoma cell line was a gift from Dr. Andrew Koff’s laboratory at Memorial Sloan Kettering Cancer Center and has been extensively characterized in the literature (Singer et al, 2007; Barretina et al, 2010; Brill et al, 2010; Gobble et al, 2011; Miller et al, 2013; Kovatcheva et al, 2015). LS8817 cells have previously been described using the nomenclature DDLS8817 (RRID:CVCL_M814). Cells were cultured in DMEM, high glucose, no glutamine (Cat# 11960044; Gibco), supplemented with 9% fetal bovine serum (Cat# 10082147; Gibco), 1% of 100× GlutaMAX supplement (Cat# 35050061; Gibco), and 1% of 10,000 U/ml penicillin/streptomycin (Cat# 15140122; Gibco), and maintained at 37°C and 5% CO_2_. Cells were authenticated by performing STR profiling to another LS8817 isogenic cell line. Cells were passaged at a 1:3 ratio every 3 d and regularly tested for *Mycoplasma*.

Human MCF-7 cells (RRID:CVCL_0031) were cultured in in 75-cm^2^ flasks containing DMEM/F-12 (Cat# 10565018; Thermo Fisher Scientific) supplemented with GlutaMAX, 10% fetal bovine serum (Cat# F4135; Sigma-Aldrich), and 1% penicillin/streptomycin at 37°C and 5% CO_2_. Before their use, MCF7 cell identity was verified by STR profiling.

### Drug treatments

Cells were passaged at a 1:3 ratio one day before drug treatment. For samples sent for sequencing, cells were treated with either 1 μM palbociclib (Cat# S1116; Selleck Chemicals) or 100 nM doxorubicin (Cat# S1208; Selleck Chemicals), with medium changes every 2–3 d until harvest.

For NF-κB inhibition experiments, 10 μM BAY11-7082 (Cat# S2913; Selleck Chemicals) was coadministered with 1 μM palbociclib in cells that had been treated with palbociclib 3 d before harvest. For the corresponding timepoints in Doxo, cells were treated with 100 nM doxorubicin for 24 h, after which the drug was washed out. 10 μM BAY11-7082 was then added for 2–3 d before harvesting the Doxo-treated cells.

For ATMi + ATRi experiments, LS8817 cells were first treated for 3 d with 1 μM palbociclib and 100 nM doxorubicin. At day 3, cells treated with palbociclib continued to receive palbociclib with the addition of ATRi (AZD6783 at 4 μM; Cat# T3338; TargetMol), ATMi + ATRi (4 μM KU-60019; Cat# S1570; Selleck Chemicals, + 4 μM AZD6783), or equal volume of DMSO as a vehicle control. Doxorubicin was washed out after ∼3 d before the addition of DMSO, ATMi, ATRi, or ATMi+ATRi until harvest. Fresh drug-containing media were changed every 2–3 d until harvest.

Stock solutions were prepared as follows: palbociclib (10 mM in ultrapure dH_2_O, stored at −80°C), doxorubicin (1 mM in ultrapure dH_2_O, stored at −20°C), BAY11-7082 (50 mM in DMSO, stored at −80°C), KU-60019 (10 mM in DMSO, stored at −80°C), AZD6783 (10 mM in DMSO, stored at −80°C).

### Immunofluorescence

50,000–60,000 cells were plated on acid-washed glass coverslips and allowed to adhere overnight. Cells were fixed for 15 min with 4% formaldehyde followed by permeabilization with 0.1% Triton X. Blocking was done with 0.5% Tween-20 and 1% normal goat serum for 30 min–1 h at room temperature (RT).

Antibody dilutions used were as follows: ATRX (Cat# A301-045A; Bethyl), 1:2,000; macroH2A (Cat# sc-377452; Santa Cruz), 1:500; γH2AX (Cat# 05-636; Millipore), 1:1,000; 53BP1 (Cat# ab172580; Abcam), 1:1,000; NF-κB p65 (Cat# 8242; Cell Signaling Technology), 1:800.

Primary antibody incubations were done at 4°C overnight. The next day, secondary Alexa Fluor antibodies (goat anti-mouse AF647, Cat# A-11004; goat anti-rabbit AF488, Cat# A-11008; Invitrogen) were diluted 1:500 with 1:2,000 Hoechst (Cat# H3570; Thermo Fisher Scientific) and incubated for 1 h at RT. All reagents, including antibodies, were diluted in 1× PBS. All steps were followed by three washes of 1× PBS. Coverslips were mounted on homemade mounting medium (1 ml 10× PBS, 9 ml ultrapure glycerol, and 50 mg of N-propyl gallate [Cat# A10877-06; Alfa Aesar]).

All images were acquired using Nikon Eclipse Ti2-E Research Microscope System and Nikon NIS-Elements acquisition software. Approximately 100 nuclei or more were used for downstream quantification. Images requiring focus quantification were acquired using a Nikon Plan Apo Lambda 60×, 1.40 NA DIC oil immersion objective lens. Images used for quantification of NF-κB p65 nuclear localization or SA-β-gal staining were acquired using a Nikon CFI ELWD S Plan Fluor 20×, 0.45 NA phase objective lens, whereas representative NF-κB p65 images were acquired with the same 60× objective lens as above. Episcopic illumination using a Lumencor SOLA Light Engine light source and DAPI (395/25-nm excitation, 425-nm dichroic, 460/50-nm emission), FITC/GFP (470/40-nm excitation, 495-nm dichroic, 525/50-nm emission), and TX Red (560/40-nm excitation, 585-nm dichroic, 630/75-nm emission) filter sets was used for immunofluorescence imaging, whereas diascopic (brightfield) illumination was used for SA-β-gal staining. Image acquisition was performed with an Andor Zyla Plus sCMOS 4.2 MP camera. Images were postprocessed with the Fiji image processing package (ImageJ), with only brightness and contrast adjusted uniformly across all experimental conditions. For all quantitative analyses, statistical significance between conditions was assessed using Dunnett’s test for multiple pairwise comparisons.

To quantify ATRX, macroH2A, γH2AX, and 53BP1 foci in immunofluorescence images, we used a custom CellProfiler pipeline (Stirling et al, 2021). Foci and nuclei were identified using the **IdentifyPrimaryObjects** module, with the **EnhanceOrSuppressFeatures** module applied beforehand to improve focus detection. The **RelateObjects** module was then used to associate foci with their respective nuclei.

The NF-κB nuclear-to-perinuclear intensity ratio was calculated using a CellProfiler pipeline based on https://www.youtube.com/watch?v=DlQSgDIGuds (CellMorphoJSero, 2021). Briefly, **IdentifyPrimaryObjects** detected nuclei, followed by **ExpandOrShrinkObjects** to expand nuclear boundaries, and **IdentifyTertiaryObjects** to define the perinuclear region. The **MeasureObjectIntensity** module was then used to quantify intensity in nuclear and perinuclear regions. SA-β-gal+–associated nuclei were manually counted using Fiji software (Schindelin et al, 2012).

### SA-β-gal staining

Senescence-associated beta-galactosidase (SA-β-gal) staining was conducted following the manufacturer’s protocol (Cat# 9860S; Cell Signaling Technology), with all reagents diluted in 1× PBS instead of dH2O.

### Colony formation assays

Clonogenic outgrowth was performed in a 6-well plate by plating 1,600, 800, and 400 cells per well using a 2× serial dilution method. Cells were allowed to grow for 10–12 d in drug-free media, followed by staining with crystal violet (1.25 g in 100 ml methanol and 400 ml distilled water) for 10 min at RT. Plates were scanned using an Epson Perfection V600 photo scanner.

### Western blots

Cells grown in a 10-cm dish were washed once with ice-cold 1× Tris-buffered saline (TBS), then scraped into 2 ml of ice-cold 1× TBS containing 1× phosSTOP (Cat# 04906845001; MilliporeSigma) and 1× cOmplete EDTA-free Protease Inhibitor (Cat# 11836170001; MilliporeSigma). Cells were spun down at 400 g for 10 min at 4°C, before removing the supernatant. 100–200 μl of 1× Laemmli buffer (Cat# 1610747; Bio-Rad) + 50 mM dithiothreitol (DTT) + 10% glycerol + 1× phosSTOP and 1× cOmplete EDTA-free Protease Inhibitor was added to the cell pellet before boiling at 95°C for 5 min. Cell lysates were then sonicated for three cycles of 10 s on and 25 s off on the Bioruptor.

4–20% Mini-PROTEAN TGX Precast Protein Gels (Cat# 4561096; Bio-Rad) were run in 1× Tris/glycine/SDS buffer (Cat# 1610732) at 200 V for 30 min, then transferred to a nitrocellulose membrane (Cytiva, 0.1 μm pore size, Cat# 10600000) overnight at 4°C in 1× transfer buffer (20 mM Tris, 150 mM glycine, 20 % methanol) (14 V). Membranes were blocked for 30 min at RT with Intercept (TBS) Protein-Free Blocking Buffer (Cat# 927-80001; LICORbio), followed by primary antibody incubation (diluted in Intercept T20 (TBS) Antibody diluent [Cat# 927-65001; LICORbio]) overnight at 4°C. Membranes were washed, then incubated at 1:10,000 dilution with secondary antibodies (Cat# 926-32211, Cat# 926-68070; LICORbio) for 1 h at RT, and washed. Blots were then imaged on a LI-COR Odyssey M system. Each wash step occurred 3 times, 5 min each wash, in 1× TBS + 0.05% Tween-20.

The following antibodies were used: phospho-p53 (Ser15) antibody (Cat# 9284; Cell Signaling Technologies), 1:1,000; p53 antibody (Cat# 9282), 1:1,000; GAPDH (D4C6R) mouse monoclonal antibody (Cat# 97166; Cell Signaling Technologies), phospho-Chk2 (Thr68) (C13C1) rabbit monoclonal antibody (Cat# 2197; Cell Signaling Technology), 1:1,000; Chk2 (D9C6) rabbit monoclonal antibody (Cat# 6334; Cell Signaling Technology), 1:1,000.

Signal intensities of protein bands relative to the loading control were quantified following the ImageJ protocol described by (Davarinejad, 2015).

### RT–qPCR

Total RNA was extracted using Quick-RNA Microprep Kit (Cat# R1050; Zymo Research) from at least two biological replicates per condition. cDNA was synthesized with Applied Biosystems High-Capacity cDNA Reverse Transcription Kit (Cat# 4368814; Thermo Fisher Scientific) and SUPERase• In RNase Inhibitor (Cat# AM2696; Thermo Fisher Scientific). Before qPCR, cDNA was diluted to 5 ng/μl in dH2O.

qPCR was performed in triplicate in a 96-well microplate using a 15 μl reaction containing 7.5 μl PowerUp SYBR Green Master Mix (Cat# A25742; Thermo Fisher Scientific), 4 μl dH2O, 0.75 μl each of forward and reverse primers, and 1.5 μl of 5 ng/μl cDNA. Reactions were run on QuantStudio 5 Real-Time PCR System with the following untreated cycling conditions: 98°C for 30 s, followed by 40–45 cycles of 98°C for 10 s, 63°C for 30 s, and 72°C for 1 min. Cq values were normalized to the housekeeping gene *PUM1*, which was selected based on its stable expression across conditions in RNA-seq datasets.

### RNA-seq on LS8817 cells

RNA extraction was done on at least two biological replicates per condition using Quick-RNA Microprep Kit (Cat# R1050; Zymo Research). Sample quality was checked by determining RNA integrity number (RIN) via running samples through High Sensitivity RNA ScreenTape (Cat# 5067-5579; Agilent Technologies) on the TapeStation.

LS8817 cells: Samples were sent to Novogene for stranded library preparation and sequencing. cDNA libraries were made with NEBNext Ultra II Directional RNA Library Prep Kit for Illumina (Cat# E7765; New England Biolabs) and sequenced on Illumina NovaSeq 6000 Sequencing System as 150-base pair-end reads.

### RNA-seq on MCF7 cells

MCF7 cells were treated with 100 nM doxorubicin (treated for 48 h, followed by drug washout), 1 μM palbociclib, or 100 nM palbociclib + 10 nM fulvestrant. Cells were harvested at day 14, day 21, and day 18, respectively, since treatment started. RNA extraction was done using Quick-RNA Microprep Kit (Cat# R1050; Zymo Research). Total RNA was sent to Plasmidsaurus for single-end RNA sequencing.

#### Preprocessing by Plasmidsaurus

Quality of the fastq files was assessed using FastQC v0.12.1. Reads were then quality-filtered using fastp v0.24.0 with poly-X tail trimming, 3′ quality-based tail trimming, a minimum Phred quality score of 15, and a minimum length requirement of 50-bp PCR, and optical duplicates were removed using UMI-based deduplication with UMIcollapse v1.1.0. Alignment quality metrics, strand specificity, and read distribution across genomic features were assessed using RSeQC v5.0.4 and Qualimap v2.3, with results aggregated into a comprehensive quality control report using MultiQC v1.32.

### ATAC sequencing

ATAC libraries were generated following the Corces et al protocol for OMNI-ATAC (Corces et al, 2017). In brief, cells were treated in culture medium with DNase (Cat# LS002007; Worthington) at a final concentration of 200 U/ml at 37°C for 30 min, then washed twice with PBS at 500 RCF, RT. Approximately ∼50,000–60,000 cells were used for each transposition reaction. After permeabilization, 2.5 μl of Illumina TDE1 enzyme (Cat# 20034198; Illumina) was used for transposition at 37°C for 30 min. Libraries were PCR-amplified for 8–13 cycles with Illumina i5 or i7 barcoded primers and were cleaned up pre- and post-amplification following the manufacturer’s instructions using Zymo DNA Clean and Concentrator-5 Kit (Cat# D4014; Zymo Research). At least two technical replicates and two biological replicates per condition were sequenced. Fragment length distribution and DNA amount were quantified via TapeStation and Qubit to assess for quality before sequencing. Libraries were sequenced on Illumina NovaSeq 6000 or on NextSeq 1000 Sequencing System.

### CUT&Tag sequencing

CUT&Tag on LS8817 cells was performed following the protocol from Kaya-Okur et al (2020) and was previously described in Soroczynski et al (2024) (*Preprint*). Antibodies used include the following: anti-histone H3 (acetyl K27) antibody—ChIP Grade (ab4729; Abcam), 1:100; guinea pig anti-rabbit IgG (heavy & light chain) antibody (ABIN101961; Antibodies-online), 1:100.

### CUT&RUN sequencing

CUT&RUN in LS8817 cells was performed on macroH2A1 (Cat# ab37264, lot #1046297-1; Abcam), 1:100, following an optimized protocol by EpiCypher (version 2.2). NEBNext Ultra II DNA Library Prep Kit for Illumina was used for fragmentation, end repair and dA-tailing, adapter ligation, and PCR amplification according to the manufacturer’s instructions. AMPure XP beads were used for post-PCR cleanup. Samples were eluted in IDTE buffer.

### Sample distance and PCA for sequencing data

Distance measures between samples were calculated using the dist function on the transposed VST-normalized count matrix for ATAC-seq or rlog-normalized count matrix for RNA-seq in R. Before this, technical replicates were merged into biological replicates for ATAC-seq. The resulting distance values were plotted using pheatmap. Principal component analysis (PCA) plots were generated using DESeq2’s plotPCA function.

### RNA-sequencing preprocessing

Reads containing adapters, N > 10% (N represents the base cannot be determined), and low quality (Qscore≤5) reads were filtered out. Pseudoalignment and quantification of transcripts were performed with kallisto (Bray et al, 2016).

### Differential gene expression analysis

*LS8817:* Downstream analysis was performed in RStudio v3.6.3. Kallisto counts were imported into R using tximport v1.14.2, and differential gene expression analysis was conducted with DESeq2 1.26.0 (Love et al, 2014). Genes with fewer than 50 total counts across all samples were filtered out. Significantly differentially expressed genes were identified using a likelihood-ratio test (*Padj* < 0.05) and a Wald test (absolute log2FC ≥ 2) relative to Cycling or day 3 treatment controls. A total of 3,744 differentially expressed genes were identified and clustered into seven RNA expression groups using k-means clustering. To characterize expression patterns across treatments, genes were further categorized as “Shared-down,” “Palbo-up-regulated,” “Doxo-up-regulated,” or “Shared-up.” rlog-normalized gene expression values were visualized using the pheatmap R package, with values scaled by row to enhance comparability.

*MCF7:* Quantification of transcripts and read alignment were performed with STAR 2.7.10a. Genes with at least 1 sample with >5 counts were kept. Differential gene expression analysis was conducted with DESeq2 (Love et al, 2014), and significantly differentially expressed genes were identified using a likelihood-ratio test (Padj < 0.05).

### Identification of SASP and transcription factor target genes

#### SASP genes

We used manually curated databases (Wang et al, 2022; Gleason et al, 2024) and the SASP Atlas (Basisty et al, 2020) (511 genes). Filtering for significantly changing genes present in all three databases, we identified 277 SASP genes that were associated with either therapy-induced senescence.

#### Cell cycle genes

Significantly changing genes were annotated as Cell Cycle if they were found in one of the following databases (892 genes): Hallmark: “E2F TARGETS,” “G2M_CHECKPOINT”; Reactome: “CELL_CYCLE,” “CELL_CYCLE_CHECKPOINTS,” “CELL_CYCLE_MITOTIC”; KEGG: “CELL_CYCLE.”

#### NF-κB target genes

We used the Gilmore Lab NF-κB target gene database (https://www.bu.edu/nf-kb/gene-resources/target-genes/; Gilmore, 2026), the “TNFA SIGNALING VIA NFKB” Hallmark term, and Ingenuity Pathway Analysis (IPA) upstream regulators containing the pattern, “NFKB” (687 genes). Filtering for significantly changing genes present in all three sources, we identified 163 up-regulated NF-κB target genes across RNA clusters.

### GO enrichment analysis

GO enrichment was performed using the enricher function from the clusterProfiler package (Yu et al, 2012), using gene IDs from each RNA cluster examining MSigDB databases (Liberzon et al. 2011, 2015). Default settings of *P*-value cutoff = 0.05 and *P-*adjust method = “BH” were used. Background gene set for GO enrichment was set as all genes expressed.

### GSEA

Genes were ranked based on the product of log2FC*−log10(*P*-value), from DESeq2 results. GSEA was performed on the ranked list of genes using the GSEA function from clusterProfiler package v3.14.3 using the default settings (Subramanian et al, 2005). Running enrichment score plots were generated using the gseaplot function with the setting “by = runningScore.”

### Ingenuity Pathway Analysis

To determine predicted upstream regulators, data were analyzed with the use of QIAGEN IPA (QIAGEN Inc., https://digitalinsights.qiagen.com/IPA) where the core analysis was performed on gene IDs from each RNA cluster (Krämer et al, 2014).

For all analyses, filtered upstream regulators were cross-referenced with a list of expressed genes in this cell line to ensure biological relevance. −log10(*Padj*) values for the top 5–10 enriched upstream regulators or activation scores for each condition were plotted as a heatmap using the pheatmap R package.

### ATAC-sequencing preprocessing

Adapters were trimmed using cutadapt, and reads were aligned with bowtie2 to the hg38 genome. After this, duplicate reads and reads mapping to the mitochondrial genome were removed. BAM files were converted to BEDPE files, which were then converted to tagAlign files for MACS2 to call peaks on, areas of the genome enriched for aligned sequencing reads, indicating hyperaccessibility. MACS2 parameters were -g 2.7 × 10^9^ -p 0.2 --shift −75 --extsize 150 --nomodel -B --SPMR --keep-dup all --call-summits. Reproducible peaks across technical replicates per condition were kept using Irreproducible Discovery Rate (Li et al, 2011). A master nonredundant peak set was generated using a custom iterative peak filtering script, which selects the peak with the lowest q-value generated by MACS2 from a set of overlapping peaks. Peaks were fixed to 500 bp in length, with 250 bp extending from the summit identified by MACS2 (Zhang et al, 2008). In total, we identified 215,671 nonredundant peaks. Fragment counts under peaks were counted using chromVAR’s getCounts function (Schep et al, 2017).

Normalized bigWig files were generated from merged BAM files of biological replicates for each condition using the bamCoverage function from deepTools with the following command: bamCoverage -b $INPUT -o $OUTPUT --binSize 5 --scaleFactor $SCALE --extendReads where $INPUT is the merged BAM file, and $OUTPUT is the resulting bigWig file. $SCALE is the same scale factor used to generate bigWig files for fragment ends.

### Differential ATAC-seq peak analysis

Differential peak analysis was performed using DESeq2 with the Wald test, based on pairwise comparisons of each timepoint relative to Cycling or day 3 treatment controls. Among the 215,671 unique peaks in our nonredundant master peak set, we identified 49,107 up-regulated and 42,164 down-regulated peaks across both drug treatments. Peaks were considered significant based on a Bonferroni-corrected *Padj* threshold (0.01/215,671 total peaks) and an absolute log2FC > 1 at any timepoint compared with Cycling or day 3 treatment controls. Up-regulated peaks were categorized as “Doxo-up-regulated” if they were significant in Doxo but not Palbo, “Palbo-up-regulated” if significant in Palbo but not Doxo, and “Shared-up-regulated” if significant in both treatments. The same criteria were applied for categorizing down-regulated peaks.

To visualize read counts under significantly up-regulated peaks, reads were variance-stabilizing transformed (VST), scaled by row, and ordered based on their classification as Palbo-up-regulated, Shared-up-regulated, or Doxo-up-regulated. Hierarchical clustering was applied within each drug category to improve the coherence of the heatmap structure. The same was performed for down-regulated peaks.

### ATAC-seq peak annotation

Significantly changing peaks at each timepoint relative to Cycling or day 3 treatment controls within each drug were merged into a single peak set. Peaks were annotated using the annotatePeak function from ChIPseeker with the UCSC hg38 knownGene TxDb (Yu et al, 2015).

### CUT&Tag and CUT&RUN preprocessing

#### CUT&Tag

Sequencing data were processed using a modular pipeline implemented in Nextflow (https://github.com/riscalab/NEXDEP-Nextflow_DNA_Epigenomic_Pipeline). Quality control was assessed using FastQC, and reports were aggregated using MultiQC to generate summary metrics across all samples. Reads were aligned to the reference genome using **Burrows–Wheeler Aligner.** SAM files generated from alignment were converted to sorted and indexed BAM files using SAMtools. Alignment statistics, including mapping rates and read counts, were calculated for each sample. Reads overlapping ENCODE blacklist regions were removed. Filtered BAM files were re-indexed before downstream analysis.

#### CUT&RUN

Cutadapt was used to trim adapters using the following code: cutadapt -a “CTG​TCT​CTT​ATA​CAC​ATC​TCC​GAG​CCC​ACG​AGA​C” -a “CTG​TCT​CTT​ATA​CAC​ATC​TGA​CGC​TGC​CGA​CGA” -o “$outfile1” -p “$outfile2” “$infile1” “$infile2.” Reads were aligned to hg38 using bowtie2 using the following parameters --end-to-end --very-sensitive --no-mixed --no-discordant --phred33 -I 10 -X 700.

Peaks were called with SEACR software (Meers et al, 2019) with parameters specified to non-normalized and stringent threshold at 0.1 for H3K27ac and 0.01 for macroH2A1. Using bedtools, BAM files were converted to BEDPE files, which were then converted to bedGraph format. bedGraph files were used as input into SEACR peak caller. bedtools intersect was used to keep peaks that were reproducible in at least 2 of 3 technical replicates before filtering for reproducible peaks across biological replicates. bedtools merge was then used to generate a nonredundant master peak set across all conditions for each histone modification. FeatureCounts was used to quantify read pairs overlapping peaks from master peak sets across conditions, with the “count all overlapping features” option enabled, using the same code for CUT&Tag.

#### Differential H3K27ac peak analysis

Peaks that had at least six samples with normalized read counts >=5 were kept. DESeq was performed on 112,034 H3K27ac peaks. The significance criteria for all H3K27ac peaks were a *Padj* value of <0.05 and an absolute log2FC value >=1 for the following conditions: Palbo versus Cycling, day 14 Doxo versus Cycling, Palbo versus day 14 Doxo.

#### Differential macroH2A1 peak analysis

Peaks with fewer than 10 normalized read counts across all samples were filtered out. DESeq was performed on 184,456 macroH2A1 peaks. Significance criteria for all mH2A1 peaks were absolute log2FC value >=1 and *Padj* < 0.01 for the following comparisons: day 28 Palbo versus Cycling, day 14 Doxo versus Cycling, day 28 Palbo versus day 14 Doxo.

### Drug-activated enhancer identification

Drug-activated enhancers were defined as regions with increased chromatin accessibility (ATAC-seq) overlapping regions of the increased H3K27ac signal. All up-regulated ATAC-seq peaks, regardless of treatment condition, that were intersected with H3K27ac peaks increased in palbociclib, doxorubicin, or both treatments and classified as Palbo-, Doxo-, or Shared-up-regulated enhancers, respectively.

### Nearby enhancer and macroH2A1-containing region identification

Genomic coordinates of genes were obtained from the comprehensive gene annotation file in GENCODE Release 41 (GRCh38.p13) (Frankish et al, 2019). For each RNA cluster of significantly changing genes, gene boundaries were extended by 50 kb upstream and downstream. Regions were then filtered to retain those overlapping with the extended genomic coordinates of genes identified in shared-up-regulated gene module (Fig 2D). In addition, nearby static peaks (peaks that did not meet the significance criteria) were identified using the same approach.

### Motif enrichment analysis

Motif enrichment analysis was performed using Analysis of Motif Enrichment (AME) from the MEME SUITE v5.0.2 (McLeay & Bailey, 2010). FASTA sequences under regions of interest were extracted using the Biostrings R package (Pages et al, 2025). Enriched motifs were identified using the HOCOMOCO v11 database (Kulakovskiy et al, 2018) with the following command: ame --verbose 1 --scoring avg --method fisher --hit-lo-fraction 0.25 --evalue-report-threshold 10.0 --control $CONTROL --kmer 2 $INPUT HOCOMOCOv11_core_HUMmono_meme_format.meme.

Here, $CONTROL represents the FASTA file containing sequences from background peaks, and $INPUT contains sequences from foreground peaks.

#### MEME-AME on nearby drug-activated enhancers

Drug-activated enhancers were first defined as regions showing increased accessibility (ATAC-seq) and H3K27ac signal upon treatment. Among these, enhancers located within ±50 kb of shared-up-regulated genes (Fig 2D), including those overlapping gene bodies, were selected. Static ATAC-seq peaks within the same ±50-kb regions served as the background set.

#### Up-regulated ATAC-seq peaks

All up-regulated ATAC-seq peaks across timepoints were merged into a single peak set and were subsequently classified as Palbo-Up (palbociclib-specific), Doxo-Up (doxorubicin-specific), or Shared-Up (up-regulated in both treatments). For each group, motif enrichment was performed using the union of up-regulated peaks from the other conditions as the background. Specifically, Palbo-Up peaks were compared against Shared-Up and Doxo-Up peaks combined, and the same strategy was applied for the other groups. FASTA sequences were extracted from up-regulated peaks at each timepoint relative to either Cycling (untreated) or day 3 treatment controls within each drug treatment; these were used as the foreground peak set, with the corresponding down-regulated ATAC-seq peaks serving as the background set. The same approach while switching the foreground and background sets was used to identify motifs enriched in down-regulated ATAC-seq peaks.

### Coverage plots

BAM files across all technical and biological replicates were merged and sorted by coordinate using SAMtools. Merged RPGC-normalized bigWig files for each condition were generated using the deepTools bamCoverage function with a bin size of 10 and effectiveGenomeSize of 2913022398. RPGC-normalized merged bigWig files for H3K27ac were used as input to plot signal ±1 kb of centers of ATAC-seq peaks that were considered as drug-activated enhancers. RPGC-normalized merged bigWig files for macroH2A1 were used as input to plot macroH2A1 signal 1 kb upstream and downstream of macroH2A1 peak centers. The bigWig signal was plotted within 1 kb of motifs using deepTools with the computeMatrix function (--referencePoint center) and plotHeatmap function.

### Identification of transcription factor target genes

#### P53 target genes

Significantly changing genes were cross-referenced with the curated p53 target list from Fischer and the “P53 PATHWAY” Hallmark term (473 genes), identifying 102 up-regulated p53 target genes across RNA clusters.

#### E2F target genes

We filtered significantly changing genes against the “E2F TARGETS” Hallmark term and Ingenuity Pathway Analysis (IPA) upstream regulators containing the pattern, “E2F” (291 genes), identifying 164 down-regulated E2F target genes.

### Activation scores of predicted upstream regulators

To determine the activation scores and visualize predicted relationships between upstream regulators, IPA core analysis was performed on log2FC values of gene IDs across timepoints relative to Cycling or day 3 treatment controls with a threshold set to an absolute value of log2FC = 1.

To identify differentially activated upstream regulators and compare between drugs, an IPA comparison was performed on the individual core analysis results, generating a matrix of activation scores. To filter for the top 10–12 most differentially activated upstream regulators, the sum of activation scores across timepoints within each drug was calculated. Upstream regulators with activation scores significantly higher in one treatment condition relative to the other were selected based on greatest magnitude differences between drugs. Conversely, upstream regulators significantly activated in Doxo-treated conditions but repressed in Palbo-treated conditions were selected based on an analogous threshold.

### Coverage plots

#### ATAC-seq accessibility signal plots around motifs

Tn5 insertion sites were identified as fragment ends from merged BAM files of biological replicates for each condition and saved as bigWig files. This was performed in R using a custom script. In brief, the resize and shift functions in GenomicRanges were used to retain and shift the ends of reads to +4, −5 bp to account for Tn5 insertion bias. The coverage function was used to convert the fragment ends into a RleList object. To normalize bigWig files, reads were counted in 500-bp bins tiled across the genome, and the resulting count matrix was used in DESeq to calculate size factors with the estimateSizeFactors function. Normalization was performed by multiplying the coverage by a scale factor (1/DESeq size factor) before exporting the resulting object as a bigWig.

Motif genomic coordinates were identified using position weight matrices from HOCOMOCO v11 and scanned across the hg38 genome with motifmatchr 1.8.0 R package (Kulakovskiy et al, 2018). Motifs overlapping up-regulated ATAC-seq peaks at any timepoint for each drug category (Doxo-up-regulated, Shared-up-regulated, Palbo-up-regulated) were saved as BED files and used for coverage plotting in deepTools (Ramírez et al, 2016). Tn5 insertion site coverage was plotted within 1 kb of motifs using deepTools with the computeMatrix function (--referencePoint center) and plotProfile or plotHeatmap function.

## Supplementary Material

Reviewer comments

## Data Availability

All sequencing datasets have been deposited in the Gene Expression Omnibus (GEO) database. Code supporting analysis of sequencing datasets can be found at https://github.com/yeungj234/Manuscript_Figures. Further information and request for resources and reagents should be directed to and will be fulfilled by the lead contact, Dr. Viviana Risca (vrisca@rockefeller.edu). ATAC-seq: GSE295258. RNA-seq: GSE295260. CUT&Tag: GSE295261. CUT&RUN: GSE336040.
