# Supplementary material for: CDK4/6 inhibition induces a senescence-associated secretory phenotype via delayed NF-κB activation
Source: Life Sci Alliance. 2026 Jul 9;9(9):e202603790. doi: 10.26508/lsa.202603790 (PMC13351265; doi:10.26508/lsa.202603790)

Uncropped western blots from Figure 1I

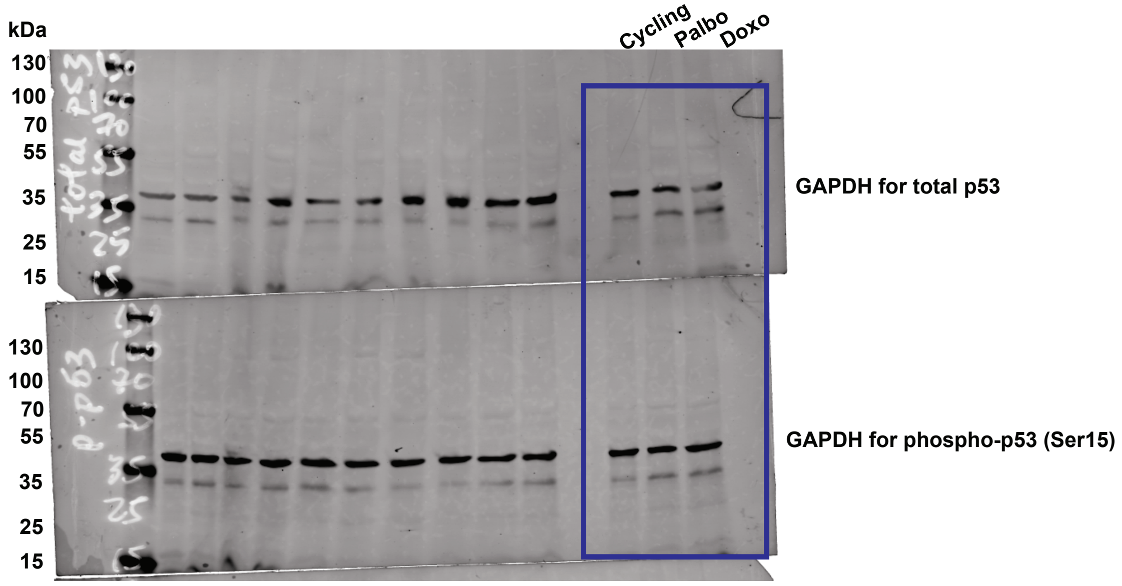

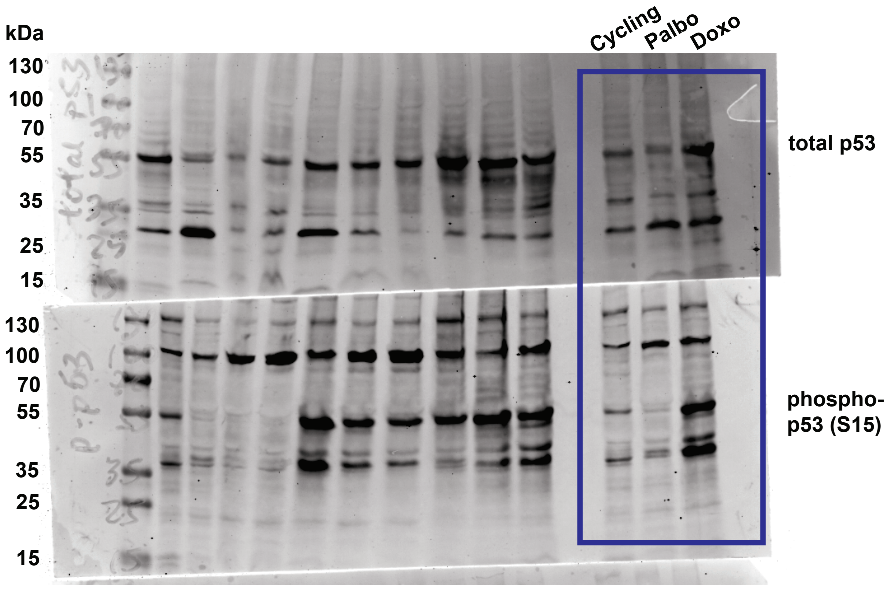

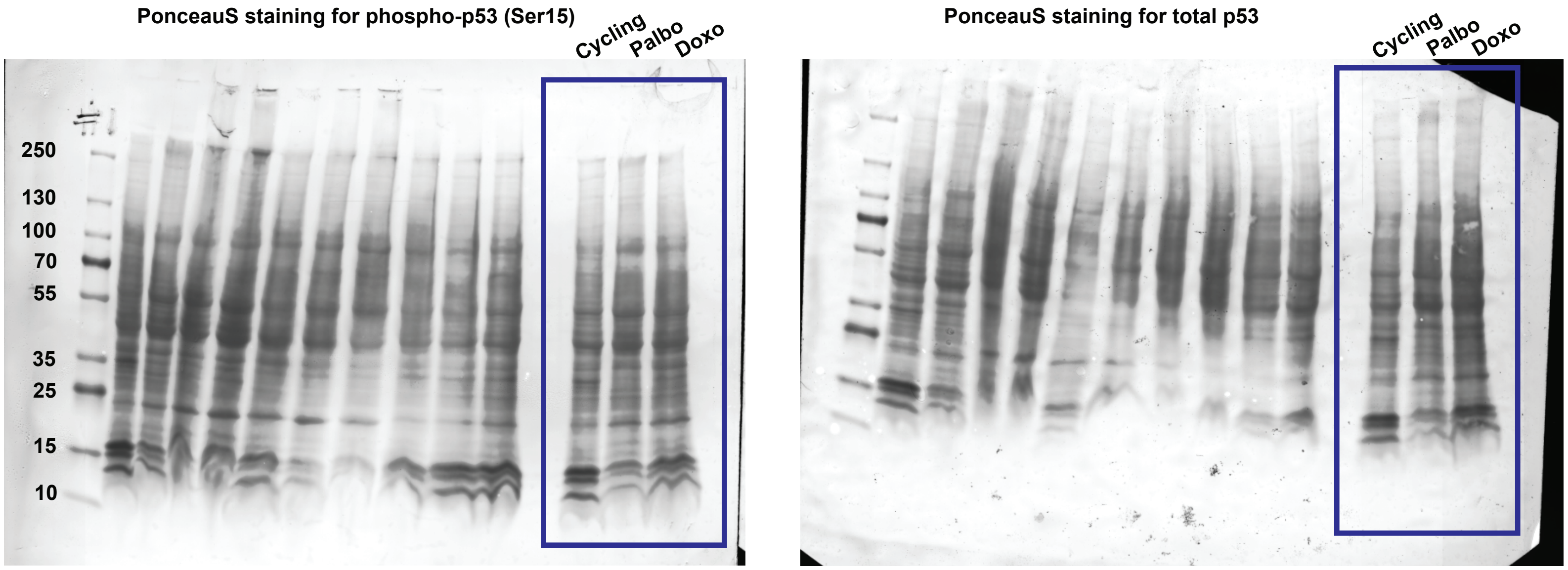

Supplement: Supplementary file 1 [file LSA-2026-03790_SdataF1.pdf]
