# Supplementary figures and images for: CDK4/6 inhibition induces a senescence-associated secretory phenotype via delayed NF-κB activation
Source: Life Sci Alliance. 2026 Jul 9;9(9):e202603790. doi: 10.26508/lsa.202603790 (PMC13351265; doi:10.26508/lsa.202603790)

Uncropped Western Blots from Figure 6A

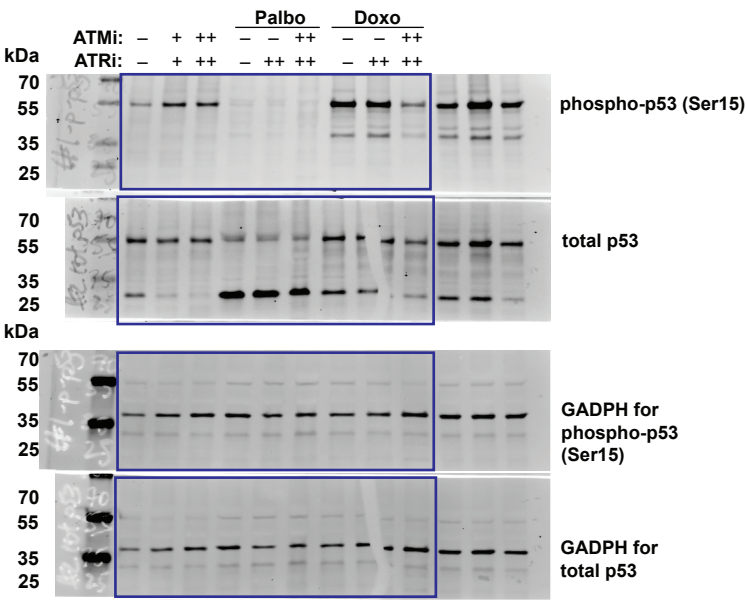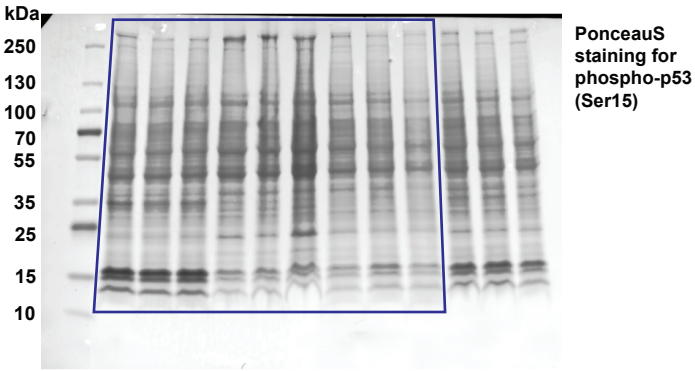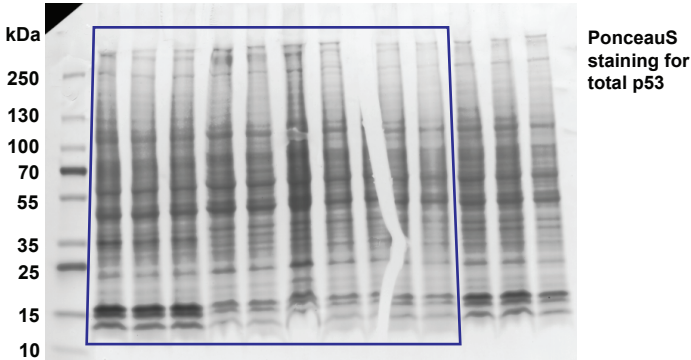

Uncropped Western Blots from Figure 6B

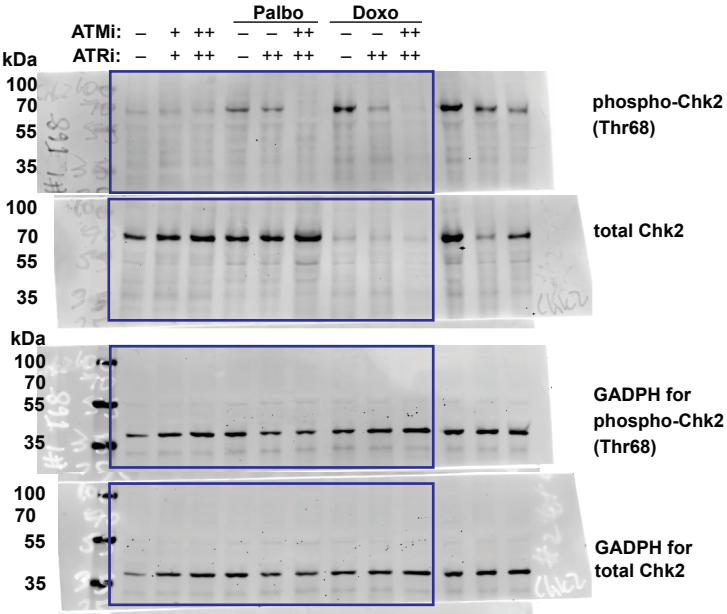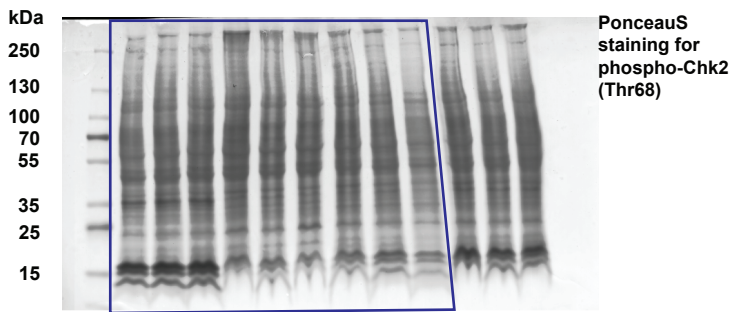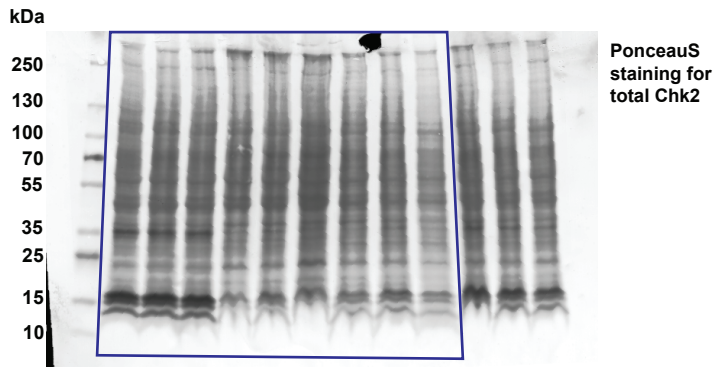

Supplement: Supplementary file 8 [file LSA-2026-03790_SdataF6.pdf]
