# Supplementary material for: CDK4/6 inhibition induces a senescence-associated secretory phenotype via delayed NF-κB activation
Source: Life Sci Alliance. 2026 Jul 9;9(9):e202603790. doi: 10.26508/lsa.202603790 (PMC13351265; doi:10.26508/lsa.202603790)
Supplement: Reviewer comments [file LSA-2026-03790_review_history.pdf]

CDK4/6 inhibition induces a senescence-associated secretory phenotype via delayed NF-κB activation

Joanna Yeung, Justin Rendleman, Lauren Anderson Westcott, Arnold Ou, Matthew Pressler, Nicole Pagane, Irene Duba, Ria Hosuru, Bat-Ider Tumenbayar, and Viviana Risca
DOI: https://doi.org/10.26508/lsa.202603790

Corresponding author(s): Viviana Risca, Rockefeller University

Review Timeline:

Submission Date:	2026-05-28
Editorial Decision:	2026-06-04
Revision Received:	2026-06-16
Accepted:	2026-06-23

Scientific Editor: Tim Fessenden

Transaction Report:

(Note: With the exception of the correction of typographical or spelling errors that could be a source of ambiguity, letters and reports are not edited. The original formatting of letters and referee reports may not be reflected in this compilation.)

Please note that the manuscript was previously reviewed at another journal and the reports were taken into account in the decision-making process at *Life Science Alliance*. Since the original reviews are not subject to Life Science Alliance's transparent review process policy, the reports and author response cannot be published.

June 4, 2026

RE: Life Science Alliance Manuscript #LSA-2026-03790-T

Viviana I. Risca
The Rockefeller University

Dear Dr. Risca,

Thank you for transferring your manuscript entitled "CDK4/6 inhibition induces a DNA damage-independent senescence-associated secretory phenotype driven by delayed activation of NF-κB" to Life Science Alliance. In accordance with our offer to publish this work, we invite you to submit a revised manuscript with text changes to resolve outstanding concerns of the reviewers at another journal. We will evaluate these changes without further input from the Reviewers.

In addition to these changes, please attend to the formatting changes detailed below in order to comport with our publication policies. We will be happy to publish this work in Life Science Alliance pending these textual and formatting changes.

MANUSCRIPT ORGANIZATION AND FORMATTING:

To avoid unnecessary delays in the acceptance and publication of your paper, please read the following information carefully. Full guidelines are available on our Instructions for Authors page, https://www.life-science-alliance.org/authors

Submission of a paper that does not conform to Life Science Alliance guidelines will delay the acceptance of your manuscript.

-Please upload all figure files individually, including the supplementary figure files; all figure legends should only appear in the main manuscript file.
-Please upload your main manuscript text as an editable doc file.
-LSA does allow supplementary methods sections; please incorporate all methods details into the main methods section.
-In the methods section, please expand the section on imaging by including the objectives, illumination, acquisition software, and post-processing steps.
-Please add ORCID ID for corresponding author - you should have received instructions on how to do so.
-Please add a Summary Blurb/Alternate Abstract in our system.
-Please add a Category for your manuscript in our system.
-Please add the X and Bluesky handles of your host institute/organization, as well as your own, and/or one of the authors, in our system.
-It is recommended to exclude figures from the manuscript text and upload them separately.
-Please clearly label the data availability section. Please ensure that all listed datasets are publicly accessible without a reviewer key.
-Please rename "COMPETING INTERESTS STATEMENT" to "Conflict of Interest."
-Please add Author Contributions to our system as well.
-Please add your main, supplementary figure, and table legends to the main manuscript text after the references section.
-Please add callouts for Figures S1B, J and S2A to your main manuscript text.

We encourage our authors to provide original source data, particularly uncropped/-processed electrophoretic blots and spreadsheets for the main figures of the manuscript. If you would like to add source data, we would welcome one PDF/Excel-file per figure for this information. These files will be linked as supplementary "Source Data" files.

It is Life Science Alliance policy that if requested, original data images must be made available to the editors. Failure to provide original images upon request will result in unavoidable delays in publication. Please ensure that you have access to all original data images prior to final submission.

If you are planning a press release on your work, please inform us immediately to allow informing our production team and

scheduling a release date.

We welcome submissions of potential cover images for the issue of LSA in which your work would appear. If you have high quality images associated with this work, please feel free to email these, with a caption, to the journal office.

LSA encourages authors to provide a 30-60 second video where the study is briefly explained. These videos will be appear embedded with the manuscript online at Life Science Alliance, and on social media to promote the published paper and authors (for examples, see https://docs.google.com/document/d/1-UWCfbE4pGcDdcgzcmiuJl2XMBJnxKYeqRvLLrLSo8s/edit?usp=sharing). Corresponding or first-authors are welcome to submit the video. Please submit only one video per manuscript. The video can be emailed to contact@life-science-alliance.org

FINAL FILES:

To upload the final version of your manuscript, please log in to your account: https://lsa.msubmit.net/cgi-bin/main.plex
You will be guided to complete the submission of your revised manuscript and to fill in all necessary information. Please get in touch in case you do not know or remember your login name.

The following items are required for acceptance.

-- An editable version of the final text (.DOC or .DOCX) is needed for copyediting (no PDFs).

-- High-resolution figure, supplementary figure and video files uploaded as individual files: See our detailed guidelines for preparing your production-ready images, https://www.life-science-alliance.org/authors

-- Summary blurb (enter in submission system): A short text summarizing in a single sentence the study (max. 200 characters including spaces). This text is used in conjunction with the titles of papers, hence should be informative and complementary to the title. It should describe the context and significance of the findings for a general readership; it should be written in the present tense and refer to the work in the third person. Author names should not be mentioned.

The license to publish form must be signed before your manuscript can be sent to production. A link to the license to publish form will be available to the corresponding author only. Please take a moment to check your funder requirements.

Reviews, decision letters, and point-by-point responses associated with peer-review at Life Science Alliance will be published, alongside the manuscript. If you do want to opt out of having the reviewer reports and your point-by-point responses displayed, please let us know immediately.

Thank you for your attention to these final processing requirements. Please revise and format the manuscript and upload materials as soon as you are able.

Thank you for this interesting contribution to the literature. We look forward to publishing your paper in Life Science Alliance.

Sincerely,

Tim Fessenden
Scientific Editor
Life Science Alliance

June 23, 2026

RE: Life Science Alliance Manuscript #LSA-2026-03790-TR

Dr. Viviana I. Risca
Rockefeller University
Laboratory of Genome Architecture and Dynamics
1230 York Ave
New York, NY 10065

Dear Dr. Risca,

Thank you for submitting your Research Article entitled "CDK4/6 inhibition induces a senescence-associated secretory phenotype via delayed NF-κB activation". We are glad to see the text changes previously requested have been incorporated, along with the requested formatting changes. It is a pleasure to let you know that your manuscript is now accepted for publication in Life Science Alliance. Congratulations on this interesting work.

The final published version of your manuscript will be deposited by us to PubMed Central upon publication.

Your manuscript will now progress through copyediting and proofing. It is journal policy that authors provide original data upon request.

Your article will publish open access upon publication under a CC-BY license.

Reviews, decision letters, and point-by-point responses associated with peer-review at Life Science Alliance will be published, alongside the manuscript. If you do want to opt out of having the reviewer reports and your point-by-point responses displayed, please let us know immediately.

IMPORTANT: If you will be unreachable at any time, please provide us with the email address of an alternate author. Failure to respond to routine queries may lead to unavoidable delays in publication.

Scheduling details will be available from our production department. You will receive proofs shortly before the publication date. Only essential corrections can be made at the proof stage so if there are any minor final changes you wish to make to the manuscript, please let the journal office know now.

DISTRIBUTION OF MATERIALS:
Authors are required to distribute freely any materials used in experiments published in Life Science Alliance. Authors are encouraged to deposit materials used in their studies to the appropriate repositories for distribution to researchers.

You can contact the journal office with any questions, contact@life-science-alliance.org

Again, congratulations on a very nice paper. I hope you are pleased with how the manuscript was handled editorially. We look forward to future exciting submissions from your lab.

Sincerely,

Tim Fessenden
Scientific Editor
Life Science Alliance
